# Reversal of chemosensitivity and induction of cell malignancy of a non-malignant prostate cancer cell line upon extracellular vesicle exposure

**DOI:** 10.1186/1476-4598-12-118

**Published:** 2013-10-08

**Authors:** Kiriaki Panagopoulos, Sam Cross-Knorr, Christen Dillard, Dionysios Pantazatos, Michael Del Tatto, David Mills, Lisa Goldstein, Joseph Renzulli, Peter Quesenberry, Devasis Chatterjee

**Affiliations:** 1 COBRE CCRD Proteomics Core, Rhode Island Hospital, Providence, RI, USA; 2 Division of Hematology/Oncology and Department of Medicine, Rhode Island Hospital, Providence, RI, USA; 3 Department of Pathology, The Miriam Hospital, Providence, RI, USA; 4 Department of Urology, The Miriam Hospital, Providence, Rhode Island, USA

**Keywords:** Extracellular vesicles, Liquid chromatography-tandem mass spectrometry, Prostate cancer, Proteomics, Gene ontology

## Abstract

**Background:**

Extracellular vesicle (EV) trafficking is a fundamental cellular process that occurs in cells and is required for different aspects of pathophysiology. EV trafficking leads to changes in cellular function including apoptosis, angiogenesis and proliferation required for increased tumor formation.

**Results:**

We report several phenotypic changes mediated by EVs isolated from non-malignant and malignant prostate cells as well as patient biopsied prostate tumor samples. EVs can reverse the resistance of prostate cancer cells to camptothecin EVs isolated from non-malignant PrECs (Prostate Epithelial Cells) can reverse soft agar colony formation of malignant DU145 cells, with the reciprocal effect observed. Isolation of EVs from 2 Gleason grade 8 prostate cancer patients significantly induced soft agar colony formation of non-malignant PrECs. We have identified proteins via antibody and Mass spectrometry analysis that may be responsible for the phenotypic changes. Mass spectrometry analysis of protein lysates using ProteoIQ revealed protein candidates associated with gene ontology annotations that may be responsible for this phenotypic change. Ingenuity Pathway Analysis was used to identify statistically relevant canonical pathways and functions associated the protein IDs and expression values obtained using ProteoIQ. Western blot analysis confirmed the increase of 14-3-3 zeta, pRKIP and prohibitin protein levels in PrEC cells co-cultured with patient EVs. 14-3-3 proteins were also found as common proteins of 3 other Gleason grade 8 patients.

**Conclusion:**

Our study provides a rational basis to further investigate putative proteins, such as 14-3-3 and prohibitin and genetic factors that may be responsible for phenotypic changes that are associated with prostate cancer progression.

## Introduction

Prostate cancer is the most frequently diagnosed malignancy and ranks second amongst all cancers in men with an estimated 218,890 men in the United States every year and 27,350 deaths per year (American Cancer Society 2007). Patients enjoy a 5-year survival rate approaching nearly 100%. However, as evidence of the slow but steady nature of this disease, 30-40% of patients will experience a PSA recurrence within 10 years following definitive surgery or radiation therapy [1]. Patients with high risk or advanced disease or staging, or who have suffered a recurrence, historically receive treatment with androgen ablation therapy. Sometimes these failures are supplemented with salvage radiation therapy and possibly receive chemotherapy [2-5]. The progression of disease is inevitable and as a result, the median survival in advanced disease is only 18 to 20 months with an overall survival of 24 to 36 months. Therefore, there is an urgent need to identify novel genes or proteins that may be useful in selecting patients for mechanism-based therapeutics to improve clinical outcome.

Recent attention has been focused on the task of identifying soluble factors secreted by tumor cells and characterizing their paracrine activities [6-10]. In addition to soluble paracrine factors, many tumor cells also release extracellular vesicles (EVs), microvesicles (MVs) or exosomes. These vesicles are distinguished by their size (30–1000 nm) and morphology and are secreted by a variety of cell types under physiological and pathological conditions; specifically they are secreted when a multivesicular endosome fuses with the plasma membrane [11,12]. According to previous studies, EVs can contain bioactive molecules, nucleic acids, and/or proteins [13]. Interestingly, the abundance of EVs released generally correlates positively with advanced grade and stage of cancer progression [14]. Activated cells of various types are known to produce and shed membrane EVs into their surroundings. However, the mechanism triggering EV generation by cancer cells is unknown. There is mounting evidence that vesicle trafficking is a highly important process in tumorigenesis. Further evaluation of vesicle trafficking may reveal a number of targets and strategies that may be important for cancer therapeutics.

Previously, it has been shown that various aspects of cellular phenotype can be transferred from one cell type to another via EVs. Accordingly there has been a strong focus on the use of EVs as a vaccine in cancer [15]. This includes the transfer of cell surface molecules of mRNA and of apoptotic bodies. One study investigated the secretion of EVs from the human prostate cancer cell lines, DU145 and LNCaP, and showed an association with a region of frequent chromosomal deletion in metastatic disease [16]. This work suggested that EVs shed from prostate cancer cells could alter the tumor microenvironment in a manner that may promote disease progression. A recent publication has demonstrated that proteins found in PC-3 cell released EVs that are mainly involved in transport, cell organization and biogenesis, metabolic process, response to stimulus, and regulation of biological processes [17].

In this study we confirmed that EVs could be utilized as a potential mechanism of suppressing growth and reversing the cancerous phenotype. We demonstrate that we can reverse the resistance of prostate cancer cells to CPT via EVs as measured by apoptosis, cytotoxicity, and growth in soft agar. In addition, growth in soft agar, a hallmark of malignant cells, can be inhibited when DU145 cells are co-cultured with EVs isolated from non-malignant human prostate epithelial (PrEC) cells with the reciprocal result occurring with PrEC cells co-cultured with DU145 EVs. Phosphoproteomic analysis revealed the transfer of numerous proteins in our co-culture model. Two proteins of significance, Suppressor of cytokine signaling 3 (SOC3) and Signal transducer and activator of transcription 3 (STAT3) were acquired or inhibited by co-culture of PrEC EVs with DU145 cells. Similarly, we found the increase in 14-3-3 zeta/delta phosphorylated Raf kinase inhibitor protein (pRKIP) and prohibitin from EVs isolated from 2 patient samples and co-cultured with PrEC. 14-3-3 zeta/delta/eta was also found as a common protein from 3 other Gleason grade 8 patients. The link between these proteins with cell survival, apoptosis induction, and tumor promotion provide a rational basis for therapeutic intervention. Therefore our study provides the basis for examining proteins released by EVs that are associated with disease progression and phenotype switching.

## Materials and methods

**Materials** All reagents and chemicals were purchased from Sigma Chemical Co. (St. Louis, MO) unless otherwise noted. ST2461, a CPT analog, was provided by Sigma Tau (Rome, Italy). Protein quantification reagents were obtained from Bio-Rad Laboratories, Inc. (Hercules, CA). Enhanced chemiluminescence reagents and secondary mouse and rabbit horseradish peroxidase-conjugated antibodies for Western blot analysis were ordered from GE Healthcare (Arlington Heights, IL). The antibody to RKIP was purchased from Millipore (Hopkington, MA); the actin-HRP, pRKIP, SOCS3, prohibitin, 14-3-3 zeta and STAT3 antibodies were purchased from Santa Cruz Biotechnology (Santa Cruz, CA); and the PARP antibody was purchased from Invitrogen (Carlsbad, CA).

### Cells

The human prostate carcinoma cell line DU145 was purchased from ATCC (Rockville, MD). The RC1 cell line, which was derived from DU145 cells, has been described [18]. The cell lines in our lab were used between passage numbers 10–20. The cells were grown in RPMI 1640 medium and supplemented with 10% fetal calf serum, glutamine, non-essential amino acids, 100 units/ml penicillin and 50 units/ml streptomycin and cultured in a humidified incubator at 37°C containing 5% CO2. The PrECs utilized in this study were obtained from Dr. William Hahn at Dana Farber Cancer Institute, Boston, MA [19] and were gown in PrEBM media supplemented with PrEGM SingleQuots (Lonza, Walkersville, MD). PrECs were used in between passage 2 and 10.

### Western blot analysis

Total cell extracts were prepared as previously described [18]. Protein concentrations of lysates were determined using the Bradford assay kit (BioRad). Proteins were separated by SDS-PAGE and electrophoretically transferred from the gel to nitrocellulose membranes (GE Healthcare). Proteins recognized by the antibodies were detected by enhanced chemilluminescence reagents (GE Healthcare).

### Tissue collection

Consent was obtained according to the Rhode Island Hospital’s Committee on Protection of Human Subjects (Institutional Review Board) for each of the patients involved. In collaboration with our colleagues, we obtained fresh prostate tissue specimens following robotic assisted laparoscopic prostatectomy (Da Vinci Robotic Surgical System at The Miriam Hospital). In accordance with the requirements mandated by the Department of Health the active robotic surgeons have maintained a detailed database with preoperative, intraoperative and postoperative parameters recorded for each case. We isolated EVs from two patients; Patient 18: Gleason 4 + 4 = 8 with tertiary 5 pattern (high risk) prostate cancer with positive margin focally and seminal vesicle invasion (pathology stage T3b). Patient 19: Gleason 4 + 4 = 8 with tertiary 5 pattern (high risk) prostate cancer with negative margins (pathology stage T3a). Patients 13, 14, and 16 were also Gleason grade 8.

### Extracellular vesicle isolation

Extracellular vesicle isolation was conducted for five different patient cancer tissue samples with Institutional Review Board approval at Rhode Island Hospital. Tumor samples were weighed and minced with a sterile scalpel into 1-2 cm pieced as previously reported [20]. Tissue pieces were then subjected to enzymatic dissociation using 0.2% collagenase in DMEM with 10% FBS for 90 minutes at 37°C and passed sequentially through 18, 22, and 25 gauge needles followed by a 40 um cell strainer as reported. The cell suspension was washed twice with DMEM and plated into a T-75 tissue culture flask with growth media consisting of DMEM 10% EV-free FBS, 1% penicillin-streptomycin. EVs were also isolated from DU145 and PrEC cell lines. DU-145 cells were plated at 1.5 × 10^5^ cells per T75 flask, and PrECs were plated at 1.5 × 10^6^ cells per 100 mm plate. Cell cultures were maintained under the previously listed conditions, and after 7 days of culture, or approximately 5 doublings of normal and tumor tissue as previously reported, the medium from the cultured cells was removed and further processed to isolate EVs [20].

The medium for EV isolation was centrifuged at 300 × gravity for 10 minutes at 4°C. The supernatant was UCF (ultra-centrifuged) at 24,000 × g for 1 hour at 4°C. The UCF pellet was resuspended in growth medium and co-cultured (self-culture or cross-culture) with cells for 4–7 days. The UCF pellet was further processed for co-culture (see below) or the isolation of protein for phospho-protein (Kinexus) and Mass Spec analysis. The supernatant from EV isolation was used as conditioned medium (CM) and was concentrated using Amicon Ultra-15 centrifugal filter unit (Millipore, Billerica, MA). The CM was filter sterilized and used for co-culture experiments described below.

### Co-culture of prostate tissue extracellular vesicles with non- and malignant prostate epithelial cell lines

Non-malignant human PrECs and malignant DU145 prostate cells were grown in Lonza Bullet or RPMI medium, respectively supplemented with special additives (PrEC) or 10% dialyzed EV-free FBS and antibiotics (DU145). The cells were co-cultured with EVs, from normal or malignant prostate tissue. Specifically, PrECs were co-cultured with EVs from prostate tumor tissue and malignant DU145 cells with EVs derived from normal prostate tissue. PrEC cells were also co-cultured for 7 days with CM isolated from DU145 cells.

### Soft agar cloning

Following EV co-culture, cells were grown in normal growth medium. After 7 days, cells were harvested for soft agar colony formation. The lower layer of the dish contained 2 ml of 1% agarose mixed with growth media; on the top level, 0.4% agarose mixed with growth media and 0.05 – 1 × 105 cells to a final volume of 1 ml. Plates were incubated in 5% CO2 at 37°C for 2–3 weeks. Colonies were then counted and images were captured on a Olympus MT2 microscope. Each experiment was analyzed in quadruplicate by 3 different individuals.

### Kinex™ antibody microarray analysis

The Kinex™ antibody microarrays are printed in quadruplicate in 32 grids of 8 × 12 spots each on glass microscope slide-sized chips with 854 antibodies from over 20 different commercial suppliers. They include 517 pan-specific antibodies for measurement of expressions of 309 protein kinases and 218 other signaling proteins, as well as 337 phospho-site-specific antibodies [21,22]. To perform a Kinex™ analysis, extracellular vesicles were harvested as previously discussed and co-culturing was conducted with 1 × 10^6^ cells on 100 mm plates. Cells were harvested washed twice with PBS, and centrifuged at 14,000 × g for 5 minutes. Supernatant was discarded and the resulting pellet was frozen at −20°C. The lysates with 50 ug protein each from the samples were labeled with the same proprietary fluorescent dye. Each sample was separately applied to opposite sides of the antibody microarray that contains a dam to prevent mixing of the samples. Following incubation of the samples with the Kinex™ chip, the unbound proteins were washed away and the chips were scanned with a Perkin-Elmer Scan Array Express Reader. Image analysis of the TIF files that were produced was performed with ImaGene 7.0 software from BioDiscovery (El Segundo, CA). Quantification of the signal intensity of all of the detected spots revealed that the difference between duplicates was within 10% for half of all of the antibodies used.

### Protein extraction for Mass Spectrometry analysis

Protein lysates from PrECs that were co-cultured with patient EVs for 7 days and control PrECs co-cultured with PrEC EVs were obtained using a ReadyPrep Sequential Extraction Kit (Bio-Rad) and then the sequential extractions were combined and cleaned up using a ReadyPrep 2-D Clean-Up Kit (Bio-Rad). Total protein concentration was determined using a BCA protein assay kit (Thermo Scientific). Samples were then resolved using NuPAGE SDS-PAGE system (Invitrogen) (4-12% acrylamide, Bis-Tris with MES SDS Running Buffer) and stained with Gel Code Blue Stain (Thermo Scientific). Gel lanes corresponding to each sample were excised into 3 bands that covered regions of high, medium, and low molecular weight proteins to reduce sample complexity. Each band was then cut into 6 mm wide pieces and subjected to in-gel tryptic digestion, and then each fraction was washed/dehydrated twice in a 1:1 solution of 0.1 M ammonium bicarbonate (Sigma) and 100% ACN (Sigma). Disulfide bonds were reduced with 10 mM dithiothreitol (DTT)(Thermo)/0.1 M ammonium bicarbonate for 45 min at 56°C and alkylated with 55 mM iodoacetamide (IAA)(Sigma) for 30 min at room temperature in the dark, and washed/dehydrated twice as explained above followed by trypsin digestion overnight at 37°C. After trypsin digestion, peptides were extracted using 25 mM ammonium bicarbonate and 100% ACN, followed by two rounds of 5% formic acid and 100% ACN. The extracts were pooled, dried in a vacuum centrifuge, and stored at −20°C until LC/MS analysis.

### Liquid chromatography/ MS analysis of protein digests

Mass spectrometry analysis was performed at the Rhode Island Hospital Proteomics Core facility by nano-LC-ESI-MS/MS using an Ultimate3000 nano-LC system (Dionex) controlled with Chromeleon software coupled to a QSTAR XL (Applied Biosystems, Concord, Ontario, CA) mass spectrometer. Tryptic digests were fractionated by reversed-phase chromatography using a C-18 PepMap 100 column (75 μm id × 15 mm, 3 μm particle size, LC Packings/Dionex, Sunnyvale, CA) operating at a flow of 300 nL/min. A linear separation gradient applied was starting at 5% (v/v) ACN in 0.1% (v/v) formic acid (Buffer A) to 95% (v/v) ACN in 0.1% (v/v) formic acid (Buffer B) over a 40 min gradient. The column eluate was introduced directly into the mass spectrometer via ESI.

Candidate ions were selected and fragmented using a standard information dependent acquisition (IDA) method. One second MS scans (range between 350 and 1800 Thompson, Thompson (Th) = Da/z) were used to identify candidates for fragmentation during MS/MS scans. MS/MS scans (2 s; range between 150 and 1800 Th) were collected up to three times after each survey scan. In order for an ion to be considered a candidate for fragmentation it had to be assigned a charge in the range of +2 to +4.

### Data processing for protein identification and quantitation

Raw LC-MS/MS data were converted using ABSciex MS Data converter software (v1.3 beta) to mgf format for protein identification using MASCOT v2.3.2 search engine (Matrix Science, Boston, MA, USA) by searching against a non-redundant human UniProt database (April 20th, 2012, containing 87,656 protein entries) using the following parameters: tryptic peptides with up to two missed cleavage sites, peptide tolerance of 0.2 Da, fragment tolerance of 0.5 Da, instrument type: ESI-QUAD-TOF, and variable modifications: methionine oxidation.

For label-free protein quantitation and proteome comparisons, raw files were converted to mzXML format using ABSciex MS Data converter software (v1.3 beta) and uploaded along with Mascot search results in .dat format into ProteoIQ software (v.2.3.08 BIOINQUIRE Athens, GA, USA). Spectral counting and relative intensity quantification were performed using precursor ion intensities, with the following parameters: mass tolerance of 20 ppm, minimum peptide length of 6 amino acids, protein probability of 0.5, and peptide probability of 0.05. After protein set generation, the proteins were further filtered using a 0.9 protein probability and normalized according to the number of spectra in each sample. Then the proteins which the 5 Gleason grade 8 patients had in common were placed in a new protein set and were filtered using GO annotations which describe the role of a given gene in a biological process, its molecular function, and cellular component. The GO terms which were selected to filter the results are related to apoptosis, inflammation, immune response, DNA transcription, and DNA translation in order to determine the importance and behavior of these proteins in apoptotic and cell survival pathways.

Ingenuity Pathway Analysis (IPA) (Ingenuity Systems, Redwood City, CA, USA) was used to identify protein networks according to biological functions and/or diseases in the Ingenuity Pathway Knowledge Base (IPKB). The protein accession numbers and the corresponding log_2_ relative expression values were uploaded into IPA, where the log_2_ relative expression values are converted to fold change values by the software. Then using these fold change values (with a cutoff of 1.5 for up- or down- regulation) for each protein, IPA determines the statistically relevant (p < 0.05) canonical pathways and functions related to the proteins in each sample. Each pathway and function is assigned a –log (p-value) that is determined by the number of proteins present in the specific pathway or function and the statistical significance of the expression level of the protein.

### Statistical methods

All cell culture experiments were repeated at least 3 times, unless indicated otherwise, and paired t-tests were used to determine statistical significance.

## Results

### Extracellular vesicle-mediated reversal of drug resistance in prostate cancer

Chemotherapy is currently the major treatment option for castration-resistance prostate cancer. However, chemoresistance is inherent in half of all patients that receive chemotherapy, and the decline of sensitivity to therapeutic agents in patients that initially respond is inevitable [23]. Multiple cellular pathways involving apoptosis, inflammation, angiogenesis, signaling intermediaries, drug efflux pumps, and tubulin are implicated in the development of chemoresistance [23]. It has been shown that resistance to CPT in DU145 cells is due, in part, to expression of Raf kinase inhibitor protein (RKIP) [18]. We hypothesized that in addition to RKIP, resistance to CPT may be due to the release of EVs.

**Figure 1:**
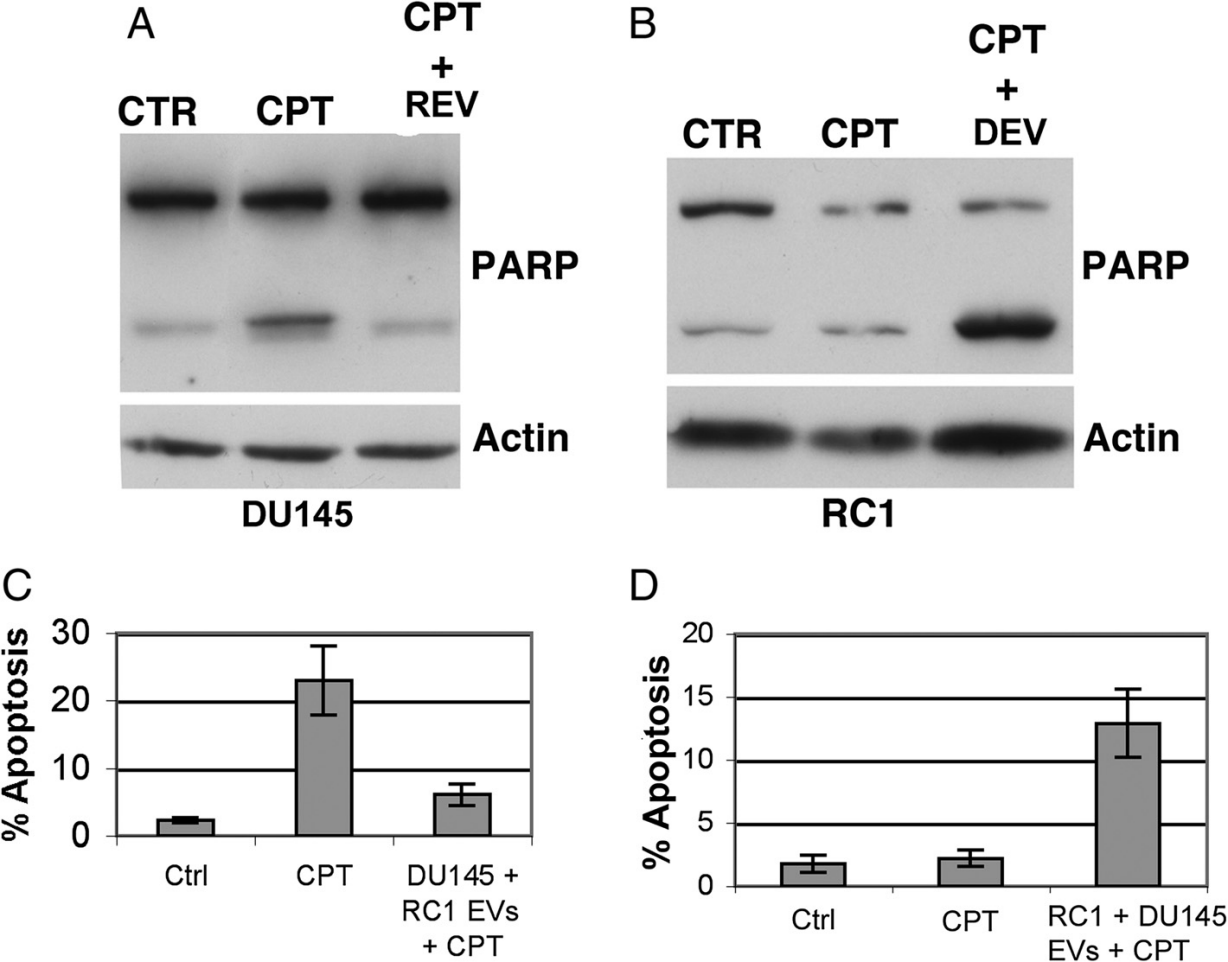
**Extracellular vesicle-mediated reversal of apoptosis resistance and sensitivity. A, B**. EVs were isolated from DU145 and RC1 cells. The EVs were resuspended in PBS. DU145 cells were co-cultured with RC1 EVs (REV) and RC1 cells were co-cultured with DU145 EVs (DEV). Non-EV and EV co-cultured cells were treated with 100 nM CPT for 24 h and examined for PARP cleavage and actin via Western blot analysis. Proteins (unless indicated 50 μg/sample was used for Western blot analysis) were separated by 10% SDS-PAGE, transferred to nitrocellulose and analyzed with antibodies to the indicated proteins. **C,D**. The same experiment from **B,C** was repeated and the samples were examined for apoptosis via propidium iodide staining using a flow cytometer. The data is the mean +/− s.d. of 2 independent experiments performed in triplicate. *Note: for all Western blots described in this Figure legend and for all other subsequent Figure legends, the exposure time used to identify the various proteins was variable.*

To investigate the effects of EV-mediated transfer of chemoresistance in prostate cancer, we studied the mechanism of resistance to camptothecin (CPT) in human prostate cancer cell lines. The DU145 cell line, a human prostate carcinoma cell line, undergoes extensive apoptosis when treated with 9-nitrocamptothecin (9NC) [18]. CPT inhibits topoisomerase I, thereby inducing single-strand breaks into the DNA molecule [24]. Conditioned media from parental DU145 cells and DU145 cells resistant to CPT (RC1 cells) were collected, ultracentrifuged, and EVs were collected for co-culture [20]. EVs isolated from DU145 cells were co-cultured with RC1 cells and EVs from RC1 cells were co-cultured with DU145 cells. After 6 days, both groups were treated with CPT, cells were harvested and analyzed for apoptosis via Poly ADP Ribose Polymerase (PARP) cleavage. Upon DNA damage PARP signals DNA repair enzymes. Treatment of DU145 with CPT indicates an increase in PARP cleavage compared to untreated control cells (CTR) indicating PARP mediated activation of DNA repair while DU145 cells co-cultured with EVs derived from RC1 cells demonstrated reduction in PARP cleavage similar to the control (Figure 1A, B). These results demonstrate EV-mediated chemoresistance of DU145 cells. DU145 cells co-cultured with RC1 EVs did not undergo apoptosis after CPT treatment, whereas the RC1 cells co-cultured with the DU145 EVs were now sensitized to the apoptosis-inducing effects of CPT (Figure 1C, D). The same experiment was repeated and the cells were analyzed for apoptosis via flow cytometry. As noted in Figure 1C, DU145 cells became resistant to CPT-induced apoptosis after co-culture with RC1 EVs. Conversely, RC1 cells underwent apoptosis after being co-cultured with DU145 cells EVs and then treated with CPT (Figure 1D). These results indicate a phenotypic shift facilitated by the co-culturing with EVs.

### Extracellular vesicle-mediated reversal of malignant prostate cancer phenotype

To better understand the phenotypic switching capacity of EVs, several strategies were applied. One of the hallmarks of malignant transformation of cells is the ability to exhibit anchorage-independent growth [25]. Therefore, we continued to examine EV-mediated phenotype changes using a non-malignant model of prostate cancer in a malignant cell line to see if the phenotype can be transferred as measured by soft agar colony formation. EVs were harvested from a malignant human prostate cancer cell line (DU145), as well as from an immortalized, non-tumorigenic prostate epithelial cell line (PrEC cells), and were collected for co-culture for vesicle characterization. EVs isolated from DU145 cells were co-cultured with PrECs and EVs from PrEC cells were co-cultured with DU145 cells. The number of EVs used for co-culture was normalized by counting the total number of EVs within a particular size of 30-1000 nm using the NanoSight NS500 (NanoSight, Wiltshire United Kingdom).

**Figure 2:**
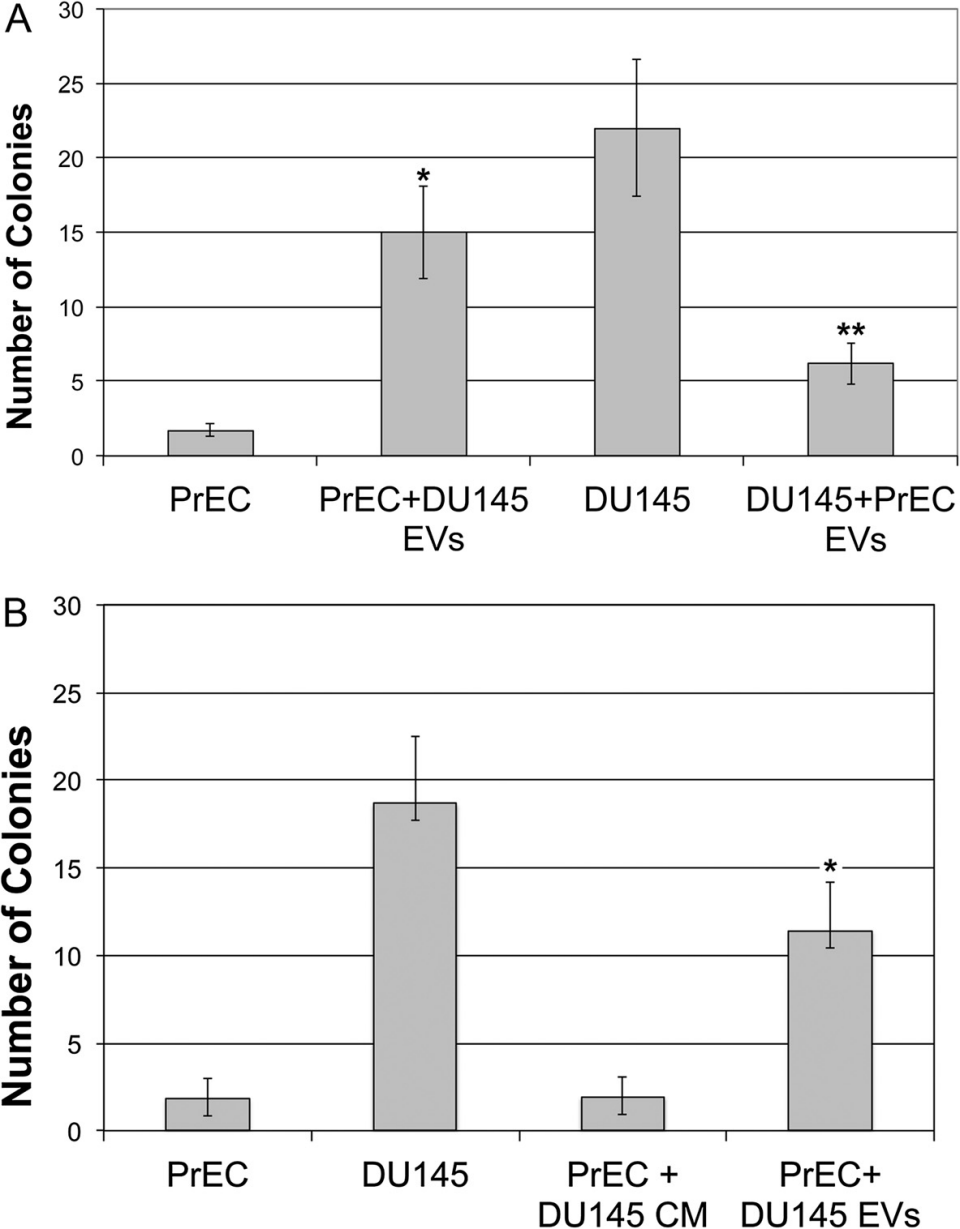
**Extracellular vesicle-mediated reversal of soft agar growth. A**, EVs were isolated from normal prostate (PrEC) and malignant (DU145) cells. PrECs were co-cultured with DU145 EVs and DU145 cells were co-cultured with PrEC EVs for 7 days. **B**, The same experiment from A was repeated with EVs and conditioned medium (CM) isolated from DU145 cells and co-cultured with PrEC cells for 7 days. 50 mls of CM was concentrated and used for the experiment. In both experiments, cells were harvested and utilized for soft agar cloning. Soft agar cloning was examined using 0.7% agarose in PBS and mixed with 1× media with 10% FBS. The top layer consisted of 0.35% agarose in PBS, 1× media with 10% FBS, and 1 x 105 cells per dish. Dishes were incubated at 37°C and 5% CO2. After two weeks, cell colonies were counted. 5 fields/dish using the 40× objective were counted and there were 5 dishes/condition. A paired t-test was performed to analyze the increase in soft agar colony formation of in A: PrEC + DU145 EVs when compared to untreated PrEC cells, * p < 0.002, and the decrease in soft agar colony formation of DU 145 + PrEC EVs when compared to untreated DU145 cells **p < 0.0004. In B: PrEC + p < 0.0003 when compared to untreated PrEC cells. *p < 0.0005.

After 7 days in culture, we measured the ability of each experimental condition (from above) to display anchorage independent growth in soft agar for 14 days. Since the malignant phenotype includes an increased ability to exhibit anchorage-independent growth, a significant increase or reduction in the number of colonies generated was viewed as a shift towards a tumorigenic phenotype or towards a normal phenotype, respectively. As shown in Figure 2, co-culture of DU145 cells with EVs isolated from PrECs prevented the colony formation in soft agar, indicating that anchorage-independent growth was significantly suppressed (p < 0.0004) in comparison to DU145 cells without EVs (Figure 2A). Remarkably, the reciprocal effect was also observed where significant changes in colony formation and anchorage independent growth in non-tumorigenic PrECs that were co-cultured with EVs isolated from DU145 cells (p < 0.0003) compared to PrECs without EVs (Figure 2A). To determine if the results we observed were a direct effect of EV co-culture with recipient cells or could be recapitulated indirectly with factors (i.e., cytokines, growth factors, etc.) released into the medium, we isolated CM from DU145 cells used for EV purification. As shown in Figure 2B, EVs isolated from DU145 cells stimulated PrEC soft agar colony formation. In contrast, CM isolated from DU145 cells did not significantly change PrEC soft agar growth (Figure 2B). This indicates that the phenotype shift we observed is a direct effect of DU145 EVs.

### Kinexus proteomic antibody array analysis of transferred proteins

To determine the proteins that are involved in or might be responsible for “phenotypic switching”, we utilized Kinexus phospho-protein microarray analysis (Vancouver, BC) in the DU145 cells co-cultured with PrEC EVs. EVs were isolated from PrECs after 7 days in culture as described [20]. PrEC EVs were then co-cultured with DU145 cells for 7 days after which the cells were harvested and the pellet sent to Kinexus Bioinformatics for analysis. The ability of EVs to elicit a phenotypic switch was therefore verified in the proteins that were transferred and analyzed. As a control a sample with DU145 cells co-cultured with DU145 EVs was also generated. Table 1 shows a portion of the results of the microarray analysis indicating the fold change in protein expression (Z score ratio).

**Table 1:** EV-mediated transfer of proteins via Kinexus phospho-protein microarray analysis in DU145 cells co-cultured with PrEC EVs

**Target protein name**	**Z-ratio (DuP, Du)**
PKCz	1.42
SOCS3	1.30
PKCm (PKD)	1.05
KAP	1.05
STAT5A	1.04
Cyclin G1	1.00
IKKa	−1.01
p38g MAPK (Erk6)	−1.02
CK1g	−1.03
MEK5 (MAP2K5)	−1.08
CDK1 (CDC2)	−1.09
STAT6	−1.09
IKKa	−1.14
RIPK1	−1.25
STAT3	−1.48
PAK3	−1.52
RSK1	−3.43

Extracellular vesicles were isolated from PrECs after 7 days in culture as described. PrEC EVs were then co-cultured with DU145 cells for 7 days after which cells were harvested and the harvested cell pellet sent to Kinexus Corporation, Vancouver, BC for analysis. As a control, a sample with DU145 cells co-cultured with DU145 EVs was also generated. These are a portion the results of the array analysis indicating the fold change in protein expression (Z score) of DU145 cells co-cultured with PrEC EVs when compared to DU145 cells co-cultured with DU145 EVs.

**Figure 3:**
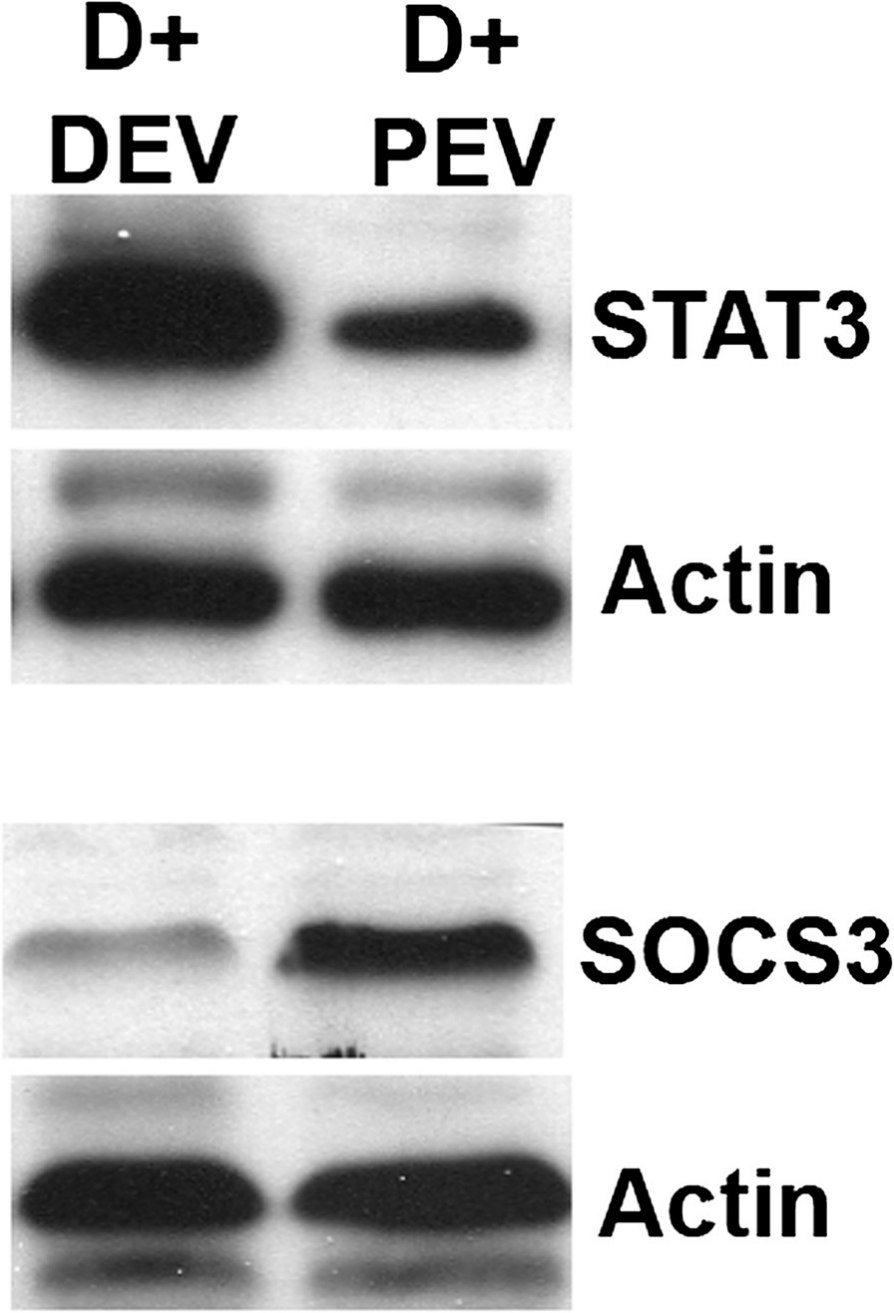
**Extracellular vesicle-mediated changes in cellular protein expression.** EVs were isolated from DU145 and PrECs and resuspended in PBS. EVs were co-cultured with DU145 cells and whole cell lysates were prepared for Western blot analysis as reported*.* Western blot analysis results show that DU145 cells co-cultured with self-EVs have enhanced expression of STAT3, while co-culture with PrEC EVs leads to increased expression of SOCS3.

To further validate these results, we confirmed the proteomic data with Western blot analysis. We performed Western blot analysis with an aliquot of the sample that was retained in the lab prior to the shipment of the samples to Kinexus. We observed a reduction STAT3 expression in DU145 cells co-cultured with self-EVs, in comparison to DU145 cells co-cultured with PrEC EVs. Further, there was a significant increase in SOCS3 expression in the DU145 cells co-cultured with PrEC EVs (Figure 3). It should be noted that we did not observe any difference in the levels of the proteins examined in parental DU145 cells when compared to DU145 cells co-cultured with DU145 EVs (data not shown). Our results implies that aberrant STAT3 signaling may be inhibited by EV release and transfer of SOCS3 or regulators of STAT3 signaling network.

### Mass spectrometry analysis of prostate cancer patient derived extracellular vesicles

**Figure 4:**
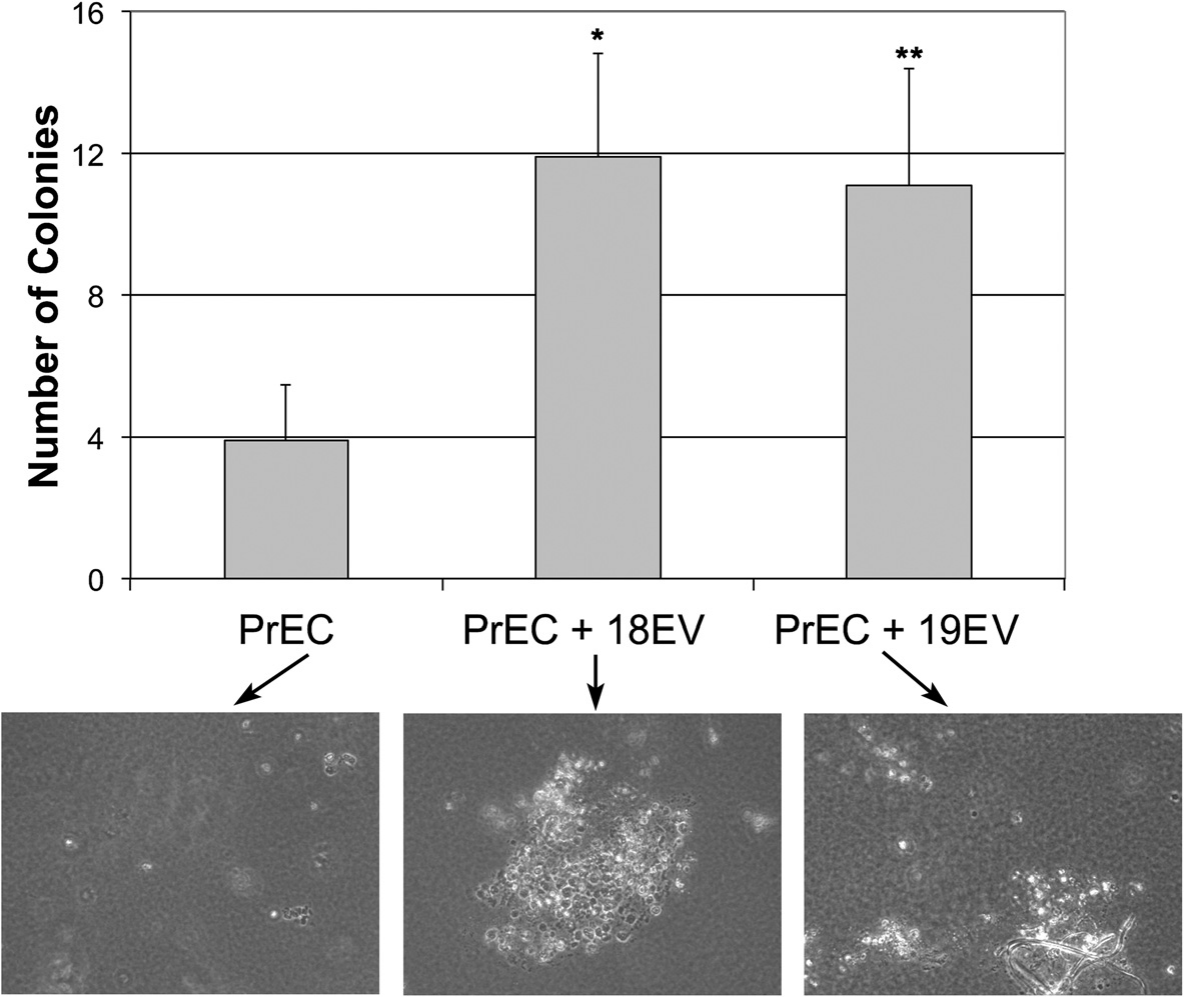
**Enhancement of soft agar growth via prostate patient-derived EVs.** EVs were isolated from 2 prostate cancer patients with Gleasons grade 8. The EVs were co-cultured with PrECs for 7 days after which soft agar growth was determined. 6 fields/dish were counted and the data represents the mean +/− s.d. of 2 independent experiments performed in triplicate. A paired t-test was performed to analyze the increase in soft agar colony formation of PrEC cells when co-cultured with EVs from patient 18, * p < 0.0005, and patient 19 **p < 0.0001 when compared to untreated PrEC cells. Note the increase in colony size in PrECs (bottom panel) co-cultured patient tumor EVs. The pictures are representative of an area of a field that was counted.

**Figure 5:**
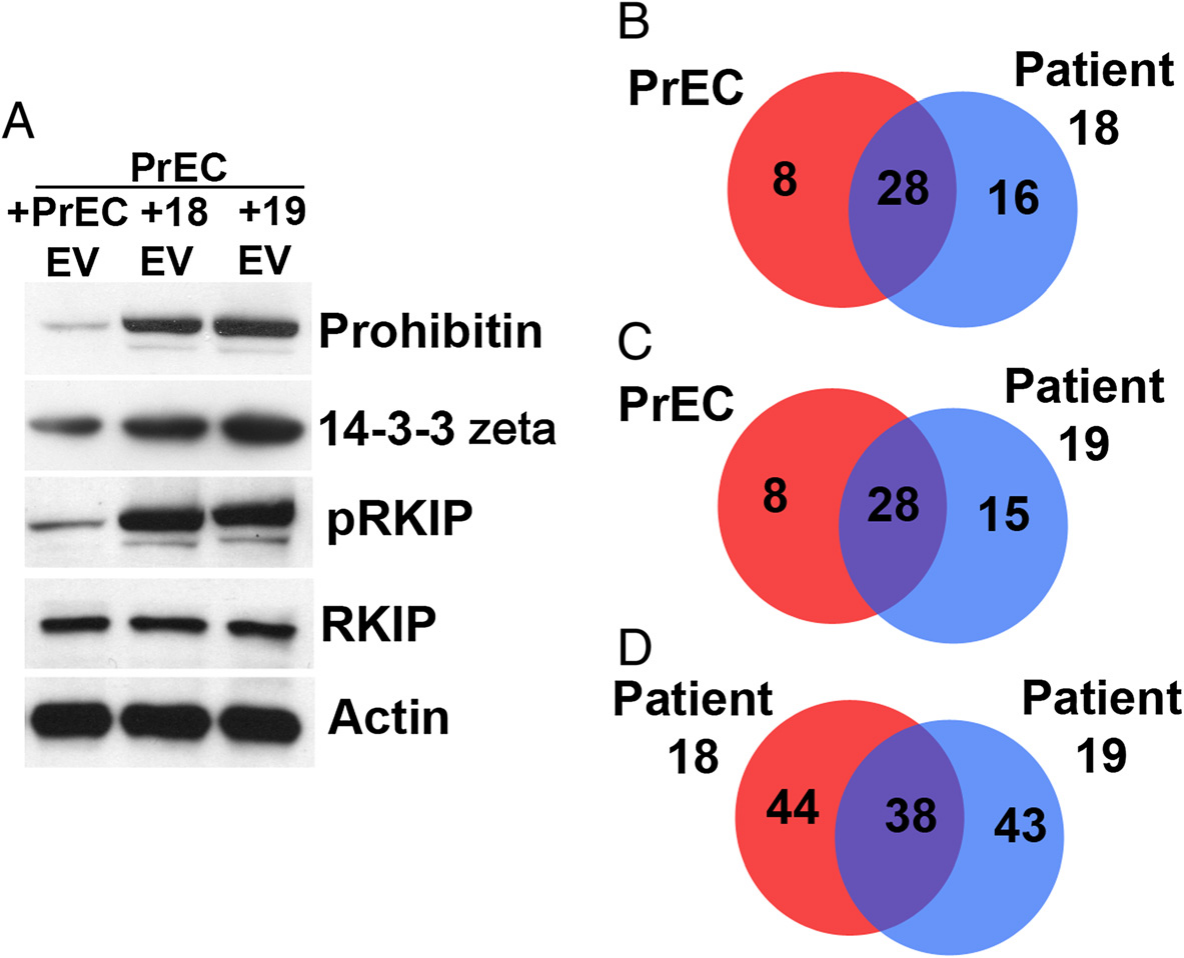
**Detection of proteins from patients EVs. A**. EVs were isolated from conditioned medium from tissue biopsied from 2 patients as described in Experimental procedures. EVs were co-cultured with PrECs for 7 days. A portion of the sample was used for mass spec analysis while the other for Western blot analysis. We examined the expression of pRKIP, RKIP, 14-3-3 zeta and actin based on the Uniprot ID data (Table 2). **B**. Venn diagram comparing the protein content of PrECs alone vs. PrECs co-cultured with Patient 18 EVs (8 proteins were unique to PrECs alone, 16 proteins were unique to PrECs co-cultured with Patient 18 EVs, and 28 proteins were found in common). **C**. Venn diagram comparing the protein content of PrECs alone vs. PrECs co-cultured with Patient 19 EVs (8 proteins were unique to PrECs alone, 15 proteins were unique to PrECs co-cultured with Patient 19 EVs, and 28 proteins were found in common). **D**. Venn diagram comparing the protein content of PrECs co-cultured with Patient 18 EVs vs. PrECs co-cultured with Patient 19 EVs (44 proteins were unique to PrECs co-cultured with Patient 18 EVs, 43 proteins were unique to PrECs co-cultured with Patient 19 EVs, and 38 proteins were found in common).

We extended our studies on DU145 and PrEC EVs and phenotype shifting to EVs derived from 2 prostate cancer patients both with Gleason grade 8. Soft agar growth was measured in PrECs after co-culture with EVs from prostate cancer patients 18 and 19. EVs from patients 18 or 19 significantly increased soft agar growth in non-malignant PrECs (p < 0.0005 and p < 0.0001, respectively) (Figure 4). A portion of the sample used for soft agar cloning was analyzed by mass spectrometry. Table 2 shows a partial list of the proteins identified in PrECs exposed to tumor-derived EVs from patients 18 and 19 as well as the log_2_ relative expression of each protein. Some 14-3-3 isoforms are associated with increased malignancy and are therapeutic targets [26] and our analysis revealed an increase of 14-3-3 zeta/delta which was confirmed by Western blot analysis (Figure 4). Also of note is the increase in pRKIP when patient 18 and 19 EVs were co-cultured with PrECs in reference to the levels of RKIP in PrECs alone. RKIP has been shown to regulate apoptosis and cell survival in prostate cancer [18]. Western blot analysis revealed that RKIP was phosphorylated after co-culture of patient 18 and 19 EVs with PrECs (Figure 5A). This result would explain, in part, our data in Figure 4 because pRKIP antagonizes the function of RKIP and allows for Raf/MAPK signaling to occur. This pathway promotes oncogenesis and cell proliferation and, presumably, soft agar growth.

**Table 2:** Comparison of relative protein expression between PrECs alone and PrECs co-cultured with patient EVs

**Accession #**	**Protein name**	**PrEC vs. PrEC + P18 EVs**		**PrEC vs. PrEC + P19 EVs**		**PrEC + P18 EVs vs. PrEC + P19 EVs**	
		**Log**_**2**_	**# Pep**	**Log**_**2**_	**# Pep**	**Log**_**2**_	**# Pep**
**P62258**	14-3-3 protein zeta	−0.64	2	0.59	2	1.23	2
**P06733**	Alpha-enolase	0.051	5	0.044	2	−0.007	2
**P04083**	Annexin A1	0.47	9	0.69	10	0.22	10
**P00403**	Cytochrome c oxidase subunit 2	0.86	4	0.82	4	−0.04	4
**P68104**	Elongation factor 1-alpha 1	0.04	8	0.39	8	0.35	8
**P30086**	PEBP1 (RKIP)	1.12	5	1.88	4	0.75	4
**P35232**	Prohibitin	0.08	2	0.41	2	0.33	2

In our analysis of the total proteome content of PrECs exposed to EVs derived from patient 18, we identified 36 protein groups in PrECs alone and 44 protein groups in PrECs with Patient 18 EVs. From these, 8 protein groups were unique to PrECs and 16 were unique in Patient 18 EVs with 28 common protein groups (Figure 5B). Exposure of PrECs with EVs from Patient 19 yielded similar results (Figure 5C). For example, Macrophage migration inhibitory factor (Uniprot ID: MIF_HUMAN) and Peptidyl-prolyl cis-trans isomerase A (Uniprot ID: PPIA_HUMAN) were found to be unique in both Patient 18 EVs and Patient 19 EVs when compared to PrECs alone. Analysis of proteome content between patients 18 and 19 yielded minimal differences between the numbers of protein groups identified in each sample indicating low patient heterogeneity (Figure 5D).

**Figure 6:**
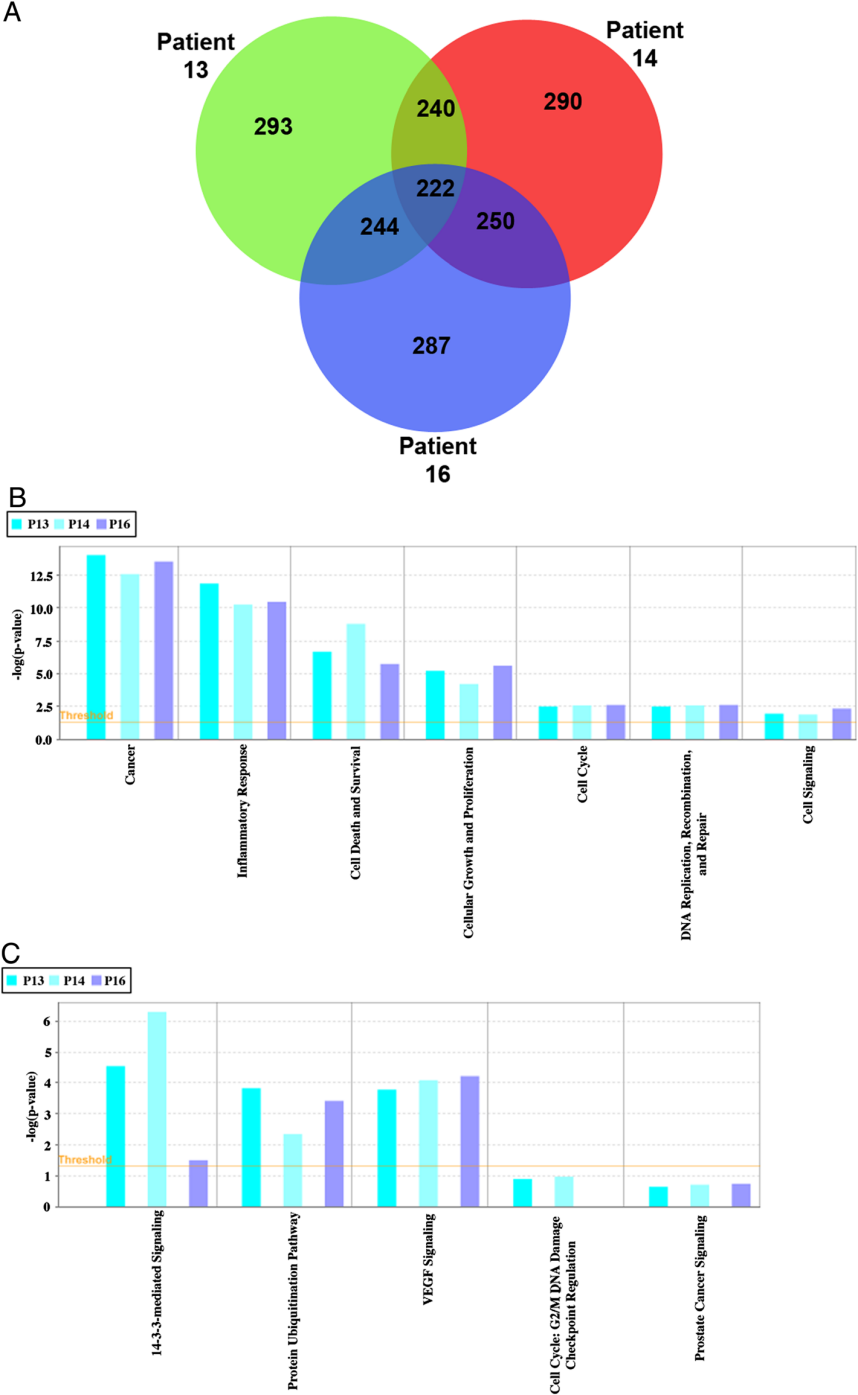
**Common proteins between patients 13, 14, and 16 and associated functions and canonical pathways.** The Venn diagram shows the common and unique proteins between patients 13, 14, and 16, **A** The bar graphs show the significance (−log(p-value)) of specific functions, **B**, and canonical pathways, **C**, in each patient. The threshold cutoff of significance is p < 0.05 (or –log = 1.3).

We examined the EV content of 3 additional Gleason grade 8 patients (Patients 13, 14, and 16) (Figure 6). The Venn diagram shows that there are 222 common proteins between these patients. The bar graph shows the functionalities listed by IPA based on the ProteoIQ protein relative expression values. The relative expression value of each protein in addition to the presence or absence of key proteins associated with that term both contribute to the significance value assigned to each function or pathway (indicated by the threshold line across the bar graph in Figure 6). The terms presented in the bar graphs were selected because they are related to cancer and/or are important topics discussed in the paper.

In addition, we compared patients 18 and 19, with patients 13, 14, and 16, and determined the common proteins between these 5 Gleason grade 8 patients. These 71 common proteins between the 5 Gleason grade 8 patients were then further filtered according to GO annotations which are related to apoptotic and cell survival pathways (seen in Tables 3, 4, 5, and 6). Some of the common proteins included: Prohibitin (Uniprot ID: PHB_HUMAN), 14-3-3 (Uniprot ID: 1433E_HUMAN) , and Annexin A1 (Uniprot ID: ANXA1_HUMAN). The importance of the proteins found in these tables is that we found proteins across all patients that are associated with apoptosis, growth and proliferation, inflammation, immune response, and DNA transcription and translation. Again with all 5 Gleason grade 8 patients was detected that 14-3-3 zeta/delta/eta was a protein in common with all 5 patients (Table 2 and Additional file 1: Table S1). This proves that there is level of homogeneity in protein content amongst these 5 Gleason 8 patients, which provides a basis for targeting these proteins to improve therapeutic methods.

**Table 3:** Apoptosis related proteins found in the common proteins between patients 13, 14, 16, 18, and 19

**Accession #**	**Protein name**
**P62258**	14-3-3 protein epsilon
**P63104**	14-3-3 protein zeta/delta
**P10809**	60 kDa heat shock protein, mitochondrial
**P11021**	78 kDa glucose-regulated protein
**O43707**	Alpha-actinin-4
**P04083**	Annexin A1
**P08758**	Annexin A5
**P27797**	Calreticulin
**P14625**	Endoplasmin
**P04406**	Glyceraldehyde-3-phosphate dehydrogenase
**P32119**	Peroxiredoxin-2
**P02545**	Prelamin-A/C
**P35232**	Prohibitin
**P14618**	Pyruvate kinase isozymes M1/M2
**P08670**	Vimentin
**P21796**	Voltage-dependent anion-selective channel protein 1
**P13010**	X-ray repair cross-complementing protein 5

Using Gene Ontology (GO) terms, the common proteins between patients 13, 14, 16, 18, and 19 were filtered according to GO terms which are related to apoptosis.

**Table 4:** Inflammation and immune response related proteins found in the common proteins between patients 13, 14, 16, 18, and 19

**Accession #**	**Protein name**
**P10809**	60 kDa heat shock protein, mitochondrial
**P04083**	Annexin A1
**P08238**	Heat shock protein HSP 90-beta
**P32119**	Peroxiredoxin-2

Using Gene Ontology (GO) terms, the common proteins between patients 13, 14, 16, 18, and 19 were filtered according to GO terms which are related to inflammation and immune response.

**Table 5:** Growth and proliferation related proteins found in the common proteins between patients 13, 14, 16, 18, and 19

**Accession #**	**Protein name**
**P62258**	14-3-3 protein epsilon
**Q04917**	14-3-3 protein eta
**P11021**	78 kDa glucose-regulated protein
**P04083**	Annexin A1
**P07355**	Annexin A2
**P06576**	ATP synthase subunit beta, mitochondrial
**P27797**	Calreticulin
**Q00610**	Clathrin heavy chain 1
**Q06830**	Peroxiredoxin-1
**P35232**	Prohibitin
**P13010**	X-ray repair cross-complementing protein 5

Using Gene Ontology (GO) terms, the common proteins between patients 13, 14, 16, 18, and 19 were filtered according to GO terms which are related to the growth and proliferation of cells.

**Table 6:** DNA transcription and translation related proteins found in the common proteins between patients 13, 14, 16, 18, and 19

**Accession #**	**Protein name**
**Q04917**	14-3-3 protein zeta
**P63104**	14-3-3 protein zeta/delta
**P06733**	Alpha-enolase
**P27824**	Calnexin
**P27797**	Calreticulin
**P04843**	Dolichyl-diphosphooligosaccharide--protein glycosyltransferase subunit 1
**P68104**	Elongation factor 1-alpha 1
**P11142**	Heat shock cognate 71 kDa protein
**P32119**	Peroxiredoxin-2
**P35232**	Prohibitin
**P68371**	Tubulin beta-4B chain
**P13010**	X-ray repair cross-complementing protein 5

Using Gene Ontology (GO) terms, the common proteins between patients 13, 14, 16, 18, and 19 were filtered according to GO terms which are related to DNA transcription and translation.

**Figure 7:**
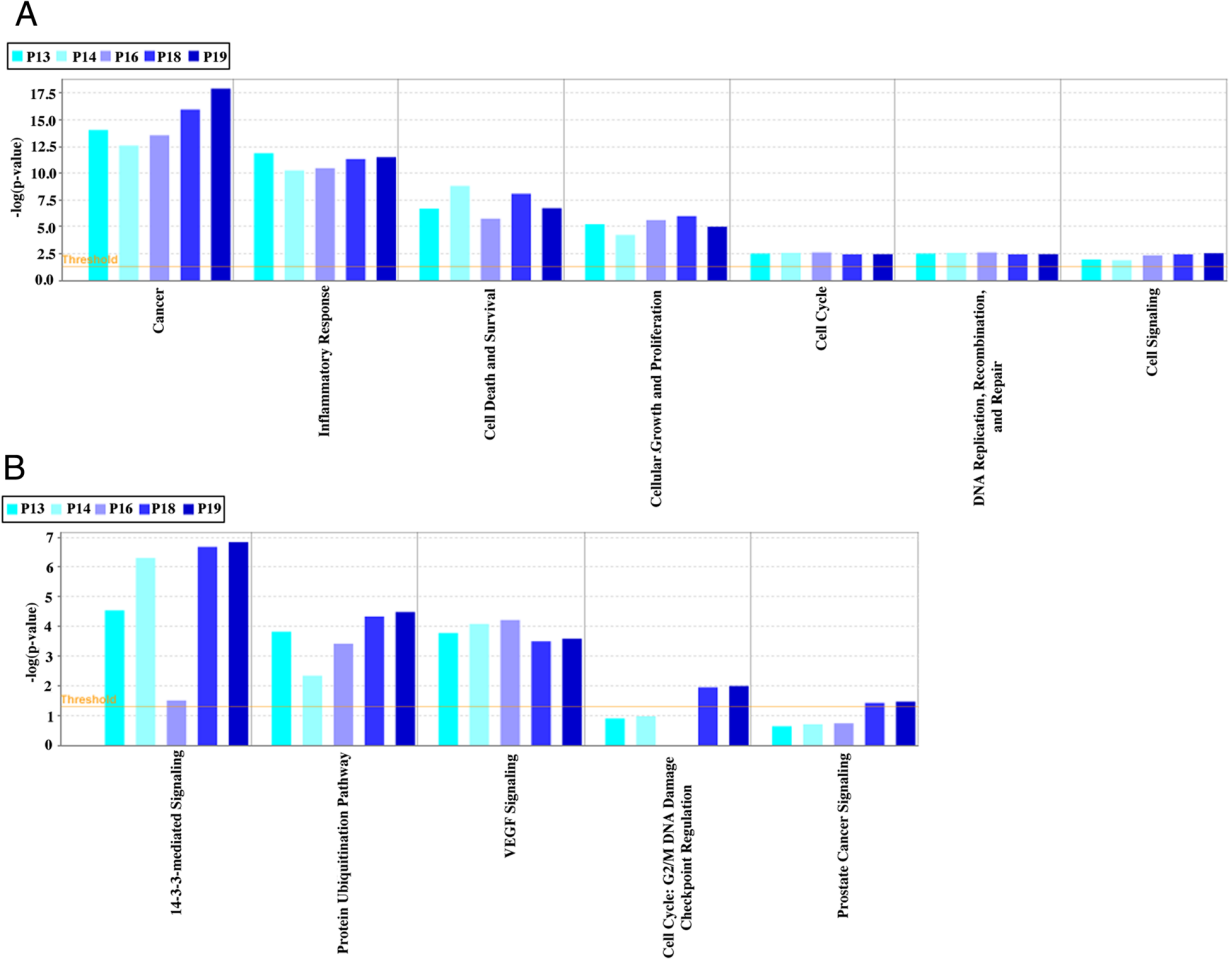
**Common proteins between patients 13, 14, 16, 18, and 19 and associated functions and canonical pathways.** IPA analysis of the 71 common proteins between patients 13, 14, 16, 18, and 19. The bar graphs show the significance (−log(p-value)) of specific functions, **A**, and canonical pathways, **B**, in each patient. The threshold cutoff of significance is p < 0.05 (or –log = 1.3).

We also analyzed this group of 71 proteins using IPA to determine whether the interaction of these proteins is similar across all of the patients. IPA showed that there is similarity in the level of significance of functions and canonical pathways related to apoptosis and cell survival across all the patients. The data sets from the 5 patients were compared using a Comparison Analysis Tool in the IPA software. The p-value, in this case, is the measure of the likelihood that the association between a set of genes in the dataset and a related function or pathway is due to random association. The cutoff value for the bar graph is set at p < 0.05 (or –log < 1.3) as indicated by the threshold line in Figure 7. So all the functions across all the patients show a statistically significant non-random association, and the majority of the pathways across all the patients also show a statistically significant non-random association. The fact that some of the canonical pathways are statistically significant in some patients but not in others can be attributed to some level of heterogeneity across patients. But for the majority of the functions and canonical pathways, the level of homogeneity in protein content and function and pathway significance can clearly be seen and can definitely prove to be a useful tool in the future development of targeted cancer therapeutics.

## Discussion

The results of this study provide direct evidence of the therapeutic potential of EVs in prostate cancer. The phenotypic changes derived by EV co-culture with normal or malignant prostate cells demonstrate the potential of EVs in the horizontal transfer of proteins. This can provide a potential opportunity for the development of new diagnostic and/or therapeutic measures. By manipulating the uptake, incorporation, and expression of exogenous material from EVs may results in phenotype switching. Horizontal gene transfer is understood to include any mechanism where genetic material is exchanged from non-parent donors to recipient cells. These methods include cell-cell interactions, such as cell fusion, and cellular interactions with mobile genetic elements, including plasmids and bacteriophages [27]. Other methods of horizontal gene transfer have been implemented in the studies of tumor progression. Gaiffe et al. illustrated that apoptotic cells can serve as DNA vectors through an endocytic process where apoptotic cells are internalized by non-specialized cells [28]. As successful gene transfer depends on *in situ* signaling, recognition, molecular triggers, and the surrounding environment, it is surprising that such unlikely elements can drive phenotype-switching, and consequently tumor progression.

In this manuscript we show that the phenotypes associated with malignant transformation and normal cell growth in prostate cells, as well as chemo-resistance/-sensitivity, can be transferred by EVs and via biopsied EVs isolated from patient tumor cells. Seemingly, exposing cells of a normal prostate phenotype with EVs from a malignant phenotype, and vice versa, leads to a transfer of biological materials between phenotypes, and therefore triggers genetic changes within the cells. Our results demonstrate that the cancer phenotype in prostate cells can be reversed or transferred via EVs. The transfer of genetic material derived from non-malignant prostate cells via EVs to a malignant cell line *in vitro* seemingly reversed the malignant transformation of the prostate cells. One unanswered question is whether the reciprocal events are occurring; if malignant-derived EVs transferred to normal cells fully facilitate a change to malignant phenotype, or if they are limited to promotion of specific hallmarks of malignant transformation. Although EVs from explant tissues (patient tissue samples) significantly increased soft agar growth in normal PrECs and malignant DU145 prostate cells, demonstrating an EV-mediated promotion of the malignant phenotype, our results are insufficient to conclude that this promotion would lead to tumorigenesis *in vivo*.

Notably, the observed genetic changes resulting from the EV-mediated, horizontal gene transfer included alterations in the expression of several clinically relevant proteins, such as RKIP. Previous studies have shown the correlation between the expression levels of RKIP and tumorigenicity in prostate cancer cells [18]. RKIP is required for human cancer cells to undergo drug-induced apoptosis, and it suppresses metastasis in prostate cancer. In regard to chemosensitivity, Chatterjee et al. showed that a rapid up-regulation of RKIP triggers apoptosis during chemotherapy treatment in drug-sensitive human prostate cells, but does not in drug-resistant cells [18]. Maximal cellular expression levels of RKIP correlated perfectly with the onset of apoptosis in chemosensitive cells, but in cells that were resistant to DNA-damaging agents, treatment with such drugs did not up-regulate RKIP expression.

We confirmed that RKIP plays a similar role in prostate tumorigenesis under our experimental conditions through assessing levels of the protein expression via mass spec. Our results indeed provided evidence that in addition to its roles in metastatic capacity and intracellular, chemotherapy-mediated apoptosis in prostate cancer cell lines, RKIP affects the chemosensitivity of prostate cells. Through packaging in EVs or by the transfer of factors that enhance protein production, RKIP expression is induced or suppressed leading to chemosensitivity or chemoresisitance, respectively. An increase in RKIP was observed from mass spec analysis (Table 2) from patient samples co-cultured with PrECs. Thus, the RKIP that is packaged in EVs provides another chemotherapy or resistance mechanism that can be targeted. Expression levels of RKIP are predictive of clinical outcome as the level of metastasis in prostate cancer decreases as the levels of RKIP expression increases. Through utilizing EVs to modify the expression levels of RKIP in prostate cancer, it is possible that the metastatic potential of tumor cells can be targeted by an EV-mediated induction of RKIP. Further, such implementation of EVs as a therapeutic strategy for prostate cancer would likely also lead to apoptosis in target cells. These findings suggest an additional role for RKIP in tumorigenesis.

Our results further indicate that the *in vitro* recipient cells of horizontally transferred genetic material, via EV co-culture, have altered expression levels of STAT3, and SOCS3. Signal transducer and activator of transcription 3 (STAT3) is a pro-survival protein responsible for the up-regulation of proteins that promote cell proliferation and growth [29,30]. During tumor development and subsequent metastatic progression, the ability of tumors to invade the lymphovascular system is mediated by STAT3 [31]. STAT proteins are substrates for the proto-oncogene c-Src, and can mediate c-Src’s biologic effects, which include androgen-induced proliferation of prostate cancer and potentially even the transition to androgen-independent growth [32,33].

Src is highly expressed in prostate cancer cell lines as well as in the majority of prostate cancer specimens [32]. Maintenance of cancer cell proliferation and survival is mediated by STAT3 even after sustained c-Src inhibition through the activation of pro-survival genes, [34]. Hence, STAT3 has been identified as an attractive target for prostate cancer, where its activity is inherently activated. Inhibition of STAT3, though disruption in activation or expression, leads to cessation of tumor cell growth and apoptosis [29]. To date, chemotherapeutic agents such as CPT have been demonstrated to down-regulate STAT3 levels. Our data suggests that EVs are immune to these limitations and can effectively eliminate the effects of STAT3 mediated signaling in prostate cancer. As shown in Figure 3, levels of STAT3 are down-regulated, and levels of SOC3 are up-regulated after the malignant DU145 cells are co-cultured with non-malignant PrEC EVs (also shown in Table 1). Suppressor of cytokine signaling 3 (SOCS3) plays a negative regulatory role in the STAT3 signaling pathway, and over-expression of the protein is adequate to prevent STAT3 activation [34]. The suppression of STAT3 suggests an endogenous epigenetic mechanism that normal, non-malignant cells produce to suppress STAT3-mediated cell survival signaling.

Utilizing 2 patients with Gleason grade 8 indicated, via mass spectrometry analysis, the enhanced RKIP and prohibitin was upregulated to non-malignant PrECs (Table 2). pRKIP is an inhibitor of RKIP function [35] and allows Raf-mediated cancer cell survival to be enhanced. Prohibitin was originally identified as a putative tumor suppressor protein that enhances p53 transcription [36-38]. A recent study has demonstrated that prohibitin proteins are necessary for the proliferation of cancer cells [39]. Thus the induction/phosphorylation of these proteins via EV-mediated transfer would promote the change, a switch in phenotype to promote prostate cell survival. Indeed our results from Figure 4 demonstrate that PrECs have acquired the ability to significantly grow in soft agar (p < 0.0005 and 0.0001) when co-cultured independently with 2 Gleason grade 8 patients EVs. Our results provide preliminary insight into putative mechanisms leading to phenotype switching of normal tissue via EVs released from malignant adjacent tissue.

14-3-3 proteins are a family of conserved regulatory molecules expressed in all eukaryotic cells. 14-3-3 proteins have the ability to bind a multitude of functionally diverse signaling proteins, including kinases, phosphatases, and transmembrane receptors. It has been demonstrated that expression of 14-3-3 proteins is significantly elevated in multiple cancers [40,41]. Our proteomic and IPA analysis determined the enhancement of 14-3-3 zeta/delta expression in our co-culture of patients 18, and 19 EVs with PrEC cells (Figure 5). In addition, these proteins along with 14-3-3 epsilon was determined to be a common protein found between patients 13,14 and 16 (Tables 3, 4 and 5). In prostate cancer models, the inhibition of PC3M cell growth was abrogated, in part, by the suppression of 14-3-3 zeta/delta [42]. It has been shown that 14-3-3zeta is an androgen-responsive gene that activates proliferation, cell survival, and androgen receptor transcriptional activity and facilitate the progression of prostate cancer [43]. Similarly, 14-3-3eta has been shown to enhance androgen- and mitogen-induced androgen receptor transcriptional activity in castration-recurrent prostate cancer [44]. In contrast, 14-3-3σ expression is down-regulated during the neoplastic transition of prostate epithelial cells [45] while there is epigenetic inactivation of 14-3-3σ occurs at an early stage of prostate tumor development [46]. 14-3-3 proteins complex with many signaling molecules, including the Raf-1 kinase [47]. 14-3-3 beta and zeta can be associated with Raf in mammalian cells and accompany Raf to the cell membrane. Therefore, 14-3-3 proteins may participate in or be required for the regulation of Raf function [48]. Raf A, B, and C activity is differentially regulated by its C-terminal and internal 14-3-3 binding domain. In this model, prohibitin, acting as scaffold protein, affected C-RAF activation in a stimulatory manner and interfered with the internal 14-3-3 binding site in C-RAF [49]. Prohibitin-mediated activation of C-Raf at membranes occurs by direct 14-3-3 displacement most probably from the internal 14-3-3 binding site [49]. We observe enhanced levels of prohibitin and 14-3-3 zeta/delta in PrEC cells after co-culture with patient 18 and 19 EVs (Figure 5). Although prohibitin may displace 14-3-3 zeta/delta from binding to Raf, 14-3-3 zeta/delta could still stimulate androgen receptor-mediated growth and prohibitin Raf-mediated MAPK signaling promoting oncogenic growth of PrEC cells. Our results suggest that 14-3-3 proteins may provide a therapeutic target in prostate cancer patients.

## Conclusions

It is highly feasible that we could target tumor progression through utilizing EVs as a therapeutic agent or tumor biomarker. The presence of EVs in cancer seemingly aids in accelerating the genetic changes necessary for tumor progression. Thus, probing the relationships between the maintenance of normal and malignant states and the import/export of proteins holds promise towards unveiling the potential diagnostic and/or predictive nature of EVs in cancer. Indeed, our detection of 14-3-3 proteins and their subsequent enhanced protein levels mediated by patient EVs provide a potential therapeutic target. Our intent is to establish EVs as indicators of therapeutic effectiveness, disease recurrence or resistance, and/or possible metastases. We are continuing our studies to identify the content of EVs derived from normal and malignant prostate tissue, using the same *in vitro* experimental approaches. We are expanding the database from which we will obtain EVs to include different grades of prostate tumor specimens from a racially and ethnically diverse population of men. Additional proteomic analysis should produce a comprehensive database of the proteins associated with EV transfer including prostate-specific trafficking genes, tumor cell surface markers, and other proteins that are specifically associated with the pathogenesis of prostate cancer. Despite the unresolved questions surrounding extracellular vesicle density, content, and transport of biological material cells between target cells, EVs could have many direct clinical applications. The ability of EVs to elicit phenotypic and genotypic changes in cancer presents an opportunity to treat cancer through blocking the transfer of genetic material by preventing EV release from cancer cells, or preventing non-malignant cells from accepting the EVs. Additionally, findings from patient-based clinical samples could be implicated in designing therapies not only directed towards the genetic/epigenetic alterations common to all prostate cancers, but also to those that are unique to each individual patients. Tailoring therapeutic intervention *a priori* form knowledge of disease course would represent a powerful clinical tool that could lead to improved outcomes and reduced recurrence of prostate cancer.

## Competing interests

The authors declare that they have no competing interests.

## Authors’ contributions

KP and SC-K contributed equally to this work. KP, SK-C, CD, MD performed all of the experiments. JR provided biopsied patient tissue. LG scored biopsied tumors. KP, SC-K, CD, DP, DM, JR, PQ and DC contributed to the result interpretation and manuscript preparation. DC, JR and PQ conceived the study. KP, DM, PQ and DC participated in its design, coordination and interpretation of the results and finalized the manuscript. All authors read and approved the final manuscript.

## Supplementary Material

Additional file 1: Table S1 List of common proteins found in Patient 13, 14, and 16 EVs. Click here for file
