# Supplementary material for: Reversal of chemosensitivity and induction of cell malignancy of a non-malignant prostate cancer cell line upon extracellular vesicle exposure
Source: Mol Cancer. 2013 Oct 8;12:118. doi: 10.1186/1476-4598-12-118 (PMC3851868; doi:10.1186/1476-4598-12-118)
Supplement: Additional file 1: Table S1 — List of common proteins found in Patient 13, 14, and 16 EVs. [file 1476-4598-12-118-S1.pdf]

Supplemental Tables

Supplemental Table 1. List of common proteins found in Patient 13, 14, and 16 EVs.

Accession #	Protein Name	P 13 EVs		P 14 EVs		P 16 EVs	
		# Unique Peptides	% Sequence Coverage	# Unique Peptides	% Sequence Coverage	# Unique Peptides	% Sequence Coverage
P62258	1433E_HUMAN 14-3-3 protein epsilon OS=Homo sapiens GN=YWHAE PE=1 SV=1	7	21.96	5	18.82	5	20.39
Q04917	1433F_HUMAN 14-3-3 protein eta OS=Homo sapiens GN=YWHAH PE=1 SV=4	7	23.17	3	10.16	2	7.32
P63104	1433Z_HUMAN 14-3-3 protein zeta/delta OS=Homo sapiens GN=YWHAZ PE=1 SV=1	7	25.71	7	33.06	5	22.86
P01009	A1AT_HUMAN Alpha-1-antitrypsin OS=Homo sapiens GN=SERPINA1 PE=1 SV=3	6	16.99	7	17.46	5	13.64
A2A274	A2A274_HUMAN Aconitase 2, mitochondrial OS=Homo sapiens GN=ACO2 PE=4 SV=1	6	11.18	3	5.84	5	7.83
A6NLG9	A6NLG9_HUMAN Biglycan OS=Homo sapiens GN=BGN PE=2 SV=1	10	35.83	10	32.9	8	31.27
A8K092	A8K092_HUMAN ATP synthase subunit alpha OS=Homo sapiens GN=ATP5A1 PE=2 SV=1	14	29.82	13	28.83	12	23.06
A8MUB1	A8MUB1_HUMAN Tubulin, alpha 1 (Testis specific), isoform CRA_a OS=Homo sapiens GN=TUBA4A PE=2 SV=1	7	21.48	6	20.79	5	15.94
P62736	ACTA_HUMAN Actin, aortic smooth muscle OS=Homo sapiens GN=ACTA2 PE=1 SV=1	20	48.54	23	51.99	17	38.46
Q562R1	ACTBL_HUMAN Beta-actin-like protein 2 OS=Homo sapiens GN=ACTBL2 PE=1 SV=2	7	16.22	11	19.15	8	19.15
O43707	ACTN4_HUMAN Alpha-actinin-4 OS=Homo sapiens GN=ACTN4 PE=1 SV=2	18	23.82	10	11.96	9	10.1
P05141	ADT2_HUMAN ADP/ATP translocase 2 OS=Homo sapiens GN=SLC25A5 PE=1 SV=7	6	20.13	3	11.41	1	3.02
P12236	ADT3_HUMAN ADP/ATP translocase 3 OS=Homo sapiens GN=SLC25A6 PE=1 SV=4	5	15.77	2	7.05	2	7.38
Q09666	AHNK_HUMAN Neuroblast differentiation-associated protein AHNAK OS=Homo sapiens GN=AHNAK PE=1 SV=2	33	7.32	31	6.93	33	8.18
P30837	AL1B1_HUMAN Aldehyde dehydrogenase X, mitochondrial OS=Homo sapiens GN=ALDH1B1 PE=1 SV=3	4	8.32	2	5.42	2	4.84
P04075	ALDOA_HUMAN Fructose-bisphosphate aldolase A OS=Homo sapiens GN=ALDOA PE=1 SV=2	8	25.27	10	33.24	4	10.99

P04083	ANXA1_HUMAN Annexin A1 OS=Homo sapiens GN=ANXA1 PE=1 SV=2	13	47.11	7	29.48	11	41.91
P07355	ANXA2_HUMAN Annexin A2 OS=Homo sapiens GN=ANXA2 PE=1 SV=2	18	44.84	15	44.54	13	37.46
P09525	ANXA4_HUMAN Annexin A4 OS=Homo sapiens GN=ANXA4 PE=1 SV=4	13	38.87	8	28.53	10	29.47
P08758	ANXA5_HUMAN Annexin A5 OS=Homo sapiens GN=ANXA5 PE=1 SV=2	11	36.25	12	37.19	10	34.06
Q16853	AOC3_HUMAN Membrane primary amine oxidase OS=Homo sapiens GN=AOC3 PE=1 SV=3	5	6.95	4	4.46	5	6.95
P02647	APOA1_HUMAN Apolipoprotein A-I OS=Homo sapiens GN=APOA1 PE=1 SV=1	1	5.99	8	36.7	9	40.07
O75947	ATP5H_HUMAN ATP synthase subunit d, mitochondrial OS=Homo sapiens GN=ATP5H PE=1 SV=3	5	42.24	5	46.58	6	32.3
P56385	ATP5I_HUMAN ATP synthase subunit e, mitochondrial OS=Homo sapiens GN=ATP5I PE=1 SV=2	1	20.29	2	24.64	1	10.14
P18859	ATP5J_HUMAN ATP synthase-coupling factor 6, mitochondrial OS=Homo sapiens GN=ATP5J PE=1 SV=1	1	12.96	3	37.04	2	30.56
P06576	ATPB_HUMAN ATP synthase subunit beta, mitochondrial OS=Homo sapiens GN=ATP5B PE=1 SV=3	12	27.6	8	19.85	7	16.82
P30049	ATPD_HUMAN ATP synthase subunit delta, mitochondrial OS=Homo sapiens GN=ATP5D PE=1 SV=2	2	13.69	2	13.69	2	13.69
P36542	ATPG_HUMAN ATP synthase subunit gamma, mitochondrial OS=Homo sapiens GN=ATP5C1 PE=1 SV=1	2	7.72	1	4.03	1	4.36
P48047	ATPO_HUMAN ATP synthase subunit O, mitochondrial OS=Homo sapiens GN=ATP5O PE=1 SV=1	4	24.88	4	23.47	3	16.43
B3KQT9	B3KQT9_HUMAN Protein disulfide isomerase family A, member 3 OS=Homo sapiens GN=PDIA3 PE=2 SV=1	14	31.25	13	29.79	18	36.88
B3KUK2	B3KUK2_HUMAN Superoxide dismutase OS=Homo sapiens GN=SOD2 PE=2 SV=1	7	38.07	2	11.93	1	7.95
B4DFL2	B4DFL2_HUMAN Isocitrate dehydrogenase [NADP] OS=Homo sapiens GN=IDH2 PE=2 SV=1	7	16.5	3	9.5	6	16
B4DIT7	B4DIT7_HUMAN Transglutaminase 2 (C polypeptide, protein-glutamine-gamma-glutamyltransferase) OS=Homo sapiens GN=TGM2 PE=2 SV=1	6	11.88	9	18.98	5	9.24

B4DPU3	B4DPU3_HUMAN Uncharacterized protein OS=Homo sapiens GN=EEF2 PE=2 SV=1	4	7.07	2	3.89	1	2.12
B4DW52	B4DW52_HUMAN Actin, beta OS=Homo sapiens GN=ACTB PE=2 SV=1	14	44.38	15	38.33	14	38.33
B4E2W0	B4E2W0_HUMAN Hydroxyacyl-CoA dehydrogenase/3-ketoacyl-CoA thiolase/enoyl-CoA hydratase (trifunctional protein), beta subunit OS=Homo sapiens GN=HADHB PE=2 SV=1	3	7.3	5	12.61	6	12.61
B7TY16	B7TY16_HUMAN Actinin alpha 1 isoform 3 OS=Homo sapiens GN=ACTN1 PE=2 SV=1	13	15.38	23	27.31	20	22.69
B7Z4V2	B7Z4V2_HUMAN Heat shock 70kDa protein 9 (mortalin) OS=Homo sapiens GN=HSPA9 PE=2 SV=1	7	13.53	4	7.97	11	23.16
B7Z7A9	B7Z7A9_HUMAN Phosphoglycerate kinase OS=Homo sapiens GN=PGK1 PE=2 SV=1	8	21.85	4	12.08	6	20.82
P80723	BASP1_HUMAN Brain acid soluble protein 1 OS=Homo sapiens GN=BASP1 PE=1 SV=2	4	27.75	3	21.59	5	44.49
P50895	BCAM_HUMAN Basal cell adhesion molecule OS=Homo sapiens GN=BCAM PE=1 SV=2	1	2.07	1	2.07	5	11.46
Q07021	C1QBP_HUMAN Complement component 1 Q subcomponent-binding protein, mitochondrial OS=Homo sapiens GN=C1QBP PE=1 SV=1	2	12.06	2	12.06	4	16.67
Q05682	CALD1_HUMAN Caldesmon OS=Homo sapiens GN=CALD1 PE=1 SV=3	3	4.67	26	29.51	17	20.68
P27797	CALR_HUMAN Calreticulin OS=Homo sapiens GN=CALR PE=1 SV=1	6	19.42	7	18.23	9	29.26
O43852	CALU_HUMAN Calumenin OS=Homo sapiens GN=CALU PE=1 SV=2	4	15.24	4	17.78	6	20.95
P27824	CALX_HUMAN Calnexin OS=Homo sapiens GN=CANX PE=1 SV=2	7	16.05	5	11.15	4	8.61
Q03135	CAV1_HUMAN Caveolin-1 OS=Homo sapiens GN=CAV1 PE=1 SV=4	3	12.92	1	5.62	3	15.17
P52907	CAZA1_HUMAN F-actin-capping protein subunit alpha-1 OS=Homo sapiens GN=CAPZA1 PE=1 SV=3	1	5.24	1	3.5	1	3.5
P61604	CH10_HUMAN 10 kDa heat shock protein, mitochondrial OS=Homo sapiens GN=HSPE1 PE=1 SV=2	5	50	5	50	5	50
P10809	CH60_HUMAN 60 kDa heat shock protein, mitochondrial OS=Homo sapiens GN=HSPD1 PE=1 SV=2	15	28.1	10	21.64	16	28.27
Q9H444	CHM4B_HUMAN Charged multivesicular body protein 4b OS=Homo sapiens GN=CHMP4B PE=1 SV=1	5	27.23	1	6.25	1	6.25

Q07065	CKAP4_HUMAN Cytoskeleton-associated protein 4 OS=Homo sapiens GN=CKAP4 PE=1 SV=2	1	1.99	2	3.82	2	4.98
Q00610	CLH1_HUMAN Clathrin heavy chain 1 OS=Homo sapiens GN=CLTC PE=1 SV=5	6	4	2	1.91	4	2.63
O00299	CLIC1_HUMAN Chloride intracellular channel protein 1 OS=Homo sapiens GN=CLIC1 PE=1 SV=4	2	8.71	1	3.32	1	4.98
P51911	CNN1_HUMAN Calponin-1 OS=Homo sapiens GN=CNN1 PE=1 SV=2	2	9.43	6	16.84	3	13.13
P02452	CO1A1_HUMAN Collagen alpha-1(I) chain OS=Homo sapiens GN=COL1A1 PE=1 SV=5	3	2.32	1	1.02	2	1.64
P01024	CO3_HUMAN Complement C3 OS=Homo sapiens GN=C3 PE=1 SV=2	4	2.83	2	1.26	1	0.6
P12109	CO6A1_HUMAN Collagen alpha-1(VI) chain OS=Homo sapiens GN=COL6A1 PE=1 SV=3	15	18.58	14	17.12	15	17.9
P12110	CO6A2_HUMAN Collagen alpha-2(VI) chain OS=Homo sapiens GN=COL6A2 PE=1 SV=4	11	10.99	11	10.99	11	9.81
P20674	COX5A_HUMAN Cytochrome c oxidase subunit 5A, mitochondrial OS=Homo sapiens GN=COX5A PE=1 SV=2	4	15.33	2	10.67	2	10.67
P10606	COX5B_HUMAN Cytochrome c oxidase subunit 5B, mitochondrial OS=Homo sapiens GN=COX5B PE=1 SV=2	1	9.3	2	15.5	4	24.81
P04080	CYTB_HUMAN Cystatin-B OS=Homo sapiens GN=CSTB PE=1 SV=2	3	45.92	1	12.24	1	12.24
D6RGG3	D6RGG3_HUMAN Collagen, type XII, alpha 1 OS=Homo sapiens GN=COL12A1 PE=4 SV=1	13	6.14	5	1.99	9	3.92
D9YZV8	D9YZV8_HUMAN Tropomyosin 1 (Alpha) isoform 7 OS=Homo sapiens GN=TPM1 PE=3 SV=1	10	27.82	29	61.62	24	51.76
O94760	DDAH1_HUMAN N(G),N(G)-dimethylarginine dimethylaminohydrolase 1 OS=Homo sapiens GN=DDAH1 PE=1 SV=3	1	3.51	3	10.53	2	6.32
P17661	DESM_HUMAN Desmin OS=Homo sapiens GN=DES PE=1 SV=3	14	28.3	32	58.72	30	53.19
Q14195-2	DPYL3_HUMAN Isoform LCRMP-4 of Dihydropyrimidinase-related protein 3 OS=Homo sapiens GN=DPYSL3	2	3.65	5	9.06	5	7.6
E7EPP3	E7EPP3_HUMAN Nidogen 2 (osteonidogen) OS=Homo sapiens GN=NID2 PE=4 SV=1	11	13.52	5	6.6	8	10.22
E7ERV9	E7ERV9_HUMAN N-acylsphingosine amidohydrolase (acid ceramidase) 1 OS=Homo sapiens GN=ASAH1 PE=4 SV=1	3	9.84	1	5.25	1	3.93
E7ESU5	E7ESU5_HUMAN Albumin OS=Homo sapiens GN=ALB PE=4 SV=2	16	24.76	25	31.39	17	22.65

E7EUQ8	E7EUQ8_HUMAN Pyruvate kinase OS=Homo sapiens GN=PKM2 PE=3 SV=1	8	17.42	1	2.15	3	5.87
E9PCV6	E9PCV6_HUMAN Collagen, type VI, alpha 3 OS=Homo sapiens GN=COL6A3 PE=4 SV=1	66	23.72	51	20.62	63	22.71
E9PGK1	E9PGK1_HUMAN Annexin OS=Homo sapiens GN=ANXA6 PE=3 SV=1	23	41.34	24	41.03	21	37.44
P40939	ECHA_HUMAN Trifunctional enzyme subunit alpha, mitochondrial OS=Homo sapiens GN=HADHA PE=1 SV=2	3	5.11	2	3.93	2	3.93
P30084	ECHM_HUMAN Enoyl-CoA hydratase, mitochondrial OS=Homo sapiens GN=ECHS1 PE=1 SV=4	2	10.34	1	7.24	4	21.03
P68104	EF1A1_HUMAN Elongation factor 1-alpha 1 OS=Homo sapiens GN=EEF1A1 PE=1 SV=1	11	22.73	6	12.12	8	16.67
P24534	EF1B_HUMAN Elongation factor 1-beta OS=Homo sapiens GN=EEF1B2 PE=1 SV=3	2	12.44	2	12.44	2	12.44
P49411	EFTU_HUMAN Elongation factor Tu, mitochondrial OS=Homo sapiens GN=TUFM PE=1 SV=2	3	9.51	2	7.3	1	3.1
P06733	ENOA_HUMAN Alpha-enolase OS=Homo sapiens GN=ENO1 PE=1 SV=2	8	21.66	8	21.66	5	14.52
P14625	ENPL_HUMAN Endoplasmin OS=Homo sapiens GN=HSP90B1 PE=1 SV=1	13	15.69	3	4.48	7	9.84
P58107	EPIPL_HUMAN Epiplakin OS=Homo sapiens GN=EPPK1 PE=1 SV=2	2	0.45	1	0.2	2	0.45
P84090	ERH_HUMAN Enhancer of rudimentary homolog OS=Homo sapiens GN=ERH PE=1 SV=1	1	10.58	1	10.58	2	16.35
P30040	ERP29_HUMAN Endoplasmic reticulum resident protein 29 OS=Homo sapiens GN=ERP29 PE=1 SV=4	5	21.84	2	7.66	5	21.84
P15311	EZRI_HUMAN Ezrin OS=Homo sapiens GN=EZR PE=1 SV=4	7	11.09	4	7.51	2	2.73
Q9UK61-2	F208A_HUMAN Isoform 2 of Protein FAM208A OS=Homo sapiens GN=FAM208A	2	0.81	2	1.62	2	1.38
F2Z393	F2Z393_HUMAN Transaldolase OS=Homo sapiens GN=TALDO1 PE=3 SV=1	4	13.84	1	3.46	2	7.86
F5H559	F5H559_HUMAN EH-domain-containing 2 OS=Homo sapiens GN=EHD2 PE=4 SV=1	3	7.32	7	16.89	2	3.56
F8VXB4	F8VXB4_HUMAN Keratin 8 OS=Homo sapiens GN=KRT8 PE=3 SV=1	14	26.42	14	26.61	14	26.42
F8VZY9	F8VZY9_HUMAN Keratin 18 OS=Homo sapiens GN=KRT18 PE=3 SV=1	9	20.97	13	27.37	13	25.06
F8WA45	F8WA45_HUMAN Gelsolin OS=Homo sapiens GN=GSN PE=4 SV=1	13	18.88	17	28.39	14	21.4

P62942	FKB1A_HUMAN Peptidyl-prolyl cis-trans isomerase FKBP1A OS=Homo sapiens GN=FKBP1A PE=1 SV=2	1	12.04	1	12.04	1	12.04
P21333	FLNA_HUMAN Filamin-A OS=Homo sapiens GN=FLNA PE=1 SV=4	48	24.03	76	35.4	69	31.36
Q14315	FLNC_HUMAN Filamin-C OS=Homo sapiens GN=FLNC PE=1 SV=3	7	2.17	37	17.17	33	15.45
P04406	G3P_HUMAN Glyceraldehyde-3-phosphate dehydrogenase OS=Homo sapiens GN=GAPDH PE=1 SV=3	5	18.21	8	23.88	4	14.03
G8JLA8	G8JLA8_HUMAN Transforming growth factor, beta-induced, 68kDa OS=Homo sapiens GN=TGFBI PE=4 SV=1	5	8.35	6	10.69	10	16.25
P11021	GRP78_HUMAN 78 kDa glucose-regulated protein OS=Homo sapiens GN=HSPA5 PE=1 SV=2	18	33.18	18	35.47	21	37.77
P09211	GSTP1_HUMAN Glutathione S-transferase P OS=Homo sapiens GN=GSTP1 PE=1 SV=2	5	29.05	1	9.52	1	3.33
H0YG33	H0YG33_HUMAN Heat shock 70kDa protein 1B OS=Homo sapiens GN=HSPA1B PE=3 SV=1	10	18.22	11	17.51	10	16.95
H0YI43	H0YI43_HUMAN Myosin, light chain 6, alkali, smooth muscle and non-muscle (Fragment) OS=Homo sapiens GN=MYL6 PE=4 SV=1	2	26.51	4	44.58	5	49.4
Q8IUE6	H2A2B_HUMAN Histone H2A type 2-B OS=Homo sapiens GN=HIST2H2AB PE=1 SV=3	1	6.92	2	12.31	1	6.92
P62805	H4_HUMAN Histone H4 OS=Homo sapiens GN=HIST1H4A PE=1 SV=2	4	38.83	5	50.49	4	38.83
P69905	HBA_HUMAN Hemoglobin subunit alpha OS=Homo sapiens GN=HBA1 PE=1 SV=2	7	42.96	5	36.62	3	23.94
P68871	HBB_HUMAN Hemoglobin subunit beta OS=Homo sapiens GN=HBB PE=1 SV=2	7	55.1	6	46.94	7	55.1
Q1KMD3	HNRL2_HUMAN Heterogeneous nuclear ribonucleoprotein U-like protein 2 OS=Homo sapiens GN=HNRNPUL2 PE=1 SV=1	1	1.74	2	3.61	2	3.61
P07900	HS90A_HUMAN Heat shock protein HSP 90-alpha OS=Homo sapiens GN=HSP90AA1 PE=1 SV=5	7	10.38	2	2.87	5	6.97
P08238	HS90B_HUMAN Heat shock protein HSP 90-beta OS=Homo sapiens GN=HSP90AB1 PE=1 SV=4	10	15.19	2	2.9	5	7.18
P11142	HSP7C_HUMAN Heat shock cognate 71 kDa protein OS=Homo sapiens GN=HSPA8 PE=1 SV=1	12	23.37	12	25.54	11	20.74

P04792	HSPB1_HUMAN Heat shock protein beta-1 OS=Homo sapiens GN=HSPB1 PE=1 SV=2	8	38.54	7	33.66	8	38.54
O75874	IDHC_HUMAN Isocitrate dehydrogenase [NADP] cytoplasmic OS=Homo sapiens GN=IDH1 PE=1 SV=2	3	9.42	2	7.25	4	11.11
P01857	IGHG1_HUMAN Ig gamma-1 chain C region OS=Homo sapiens GN=IGHG1 PE=1 SV=1	2	8.79	5	21.21	3	11.21
P01834	IGKC_HUMAN Ig kappa chain C region OS=Homo sapiens GN=IGKC PE=1 SV=1	3	49.06	3	49.06	3	49.06
P46940	IQGA1_HUMAN Ras GTPase-activating-like protein IQGAP1 OS=Homo sapiens GN=IQGAP1 PE=1 SV=1	5	3.62	2	2.17	1	0.72
P56199	ITA1_HUMAN Integrin alpha-1 OS=Homo sapiens GN=ITGA1 PE=1 SV=2	11	9.92	1	1.1	1	1.1
P08648	ITA5_HUMAN Integrin alpha-5 OS=Homo sapiens GN=ITGA5 PE=1 SV=2	8	9.91	5	7.34	4	4.77
P05556	ITB1_HUMAN Integrin beta-1 OS=Homo sapiens GN=ITGB1 PE=1 SV=2	9	12.66	7	10.65	6	8.27
P13645	K1C10_HUMAN Keratin, type I cytoskeletal 10 OS=Homo sapiens GN=KRT10 PE=1 SV=6	7	12.5	3	4.45	9	15.92
P13646	K1C13_HUMAN Keratin, type I cytoskeletal 13 OS=Homo sapiens GN=KRT13 PE=1 SV=4	11	20.74	3	6.11	7	12.66
P02533	K1C14_HUMAN Keratin, type I cytoskeletal 14 OS=Homo sapiens GN=KRT14 PE=1 SV=4	13	20.13	6	12.08	10	17.37
P19012	K1C15_HUMAN Keratin, type I cytoskeletal 15 OS=Homo sapiens GN=KRT15 PE=1 SV=3	9	16.23	5	9.65	13	27.41
P08779	K1C16_HUMAN Keratin, type I cytoskeletal 16 OS=Homo sapiens GN=KRT16 PE=1 SV=4	11	24.52	5	9.3	7	12.05
Q04695	K1C17_HUMAN Keratin, type I cytoskeletal 17 OS=Homo sapiens GN=KRT17 PE=1 SV=2	23	45.6	8	18.29	16	32.64
P08727	K1C19_HUMAN Keratin, type I cytoskeletal 19 OS=Homo sapiens GN=KRT19 PE=1 SV=4	25	60	14	33.75	21	52.5
P35527	K1C9_HUMAN Keratin, type I cytoskeletal 9 OS=Homo sapiens GN=KRT9 PE=1 SV=3	3	7.38	4	9.47	7	16.85
P35908	K22E_HUMAN Keratin, type II cytoskeletal 2 epidermal OS=Homo sapiens GN=KRT2 PE=1 SV=2	7	11.42	5	7.98	11	19.09
P04264	K2C1_HUMAN Keratin, type II cytoskeletal 1 OS=Homo sapiens GN=KRT1 PE=1 SV=6	10	16.93	8	13.51	14	22.05
P13647	K2C5_HUMAN Keratin, type II cytoskeletal 5 OS=Homo sapiens GN=KRT5 PE=1 SV=3	18	30.68	12	22.37	15	25.59
P02538	K2C6A_HUMAN Keratin, type II cytoskeletal 6A OS=Homo sapiens GN=KRT6A PE=1 SV=3	20	35.64	6	11.17	8	13.83
P08729	K2C7_HUMAN Keratin, type II cytoskeletal 7 OS=Homo sapiens GN=KRT7 PE=1 SV=5	19	37.53	5	11.09	9	20.26

Q7RTS7	K2C74_HUMAN Keratin, type II cytoskeletal 74 OS=Homo sapiens GN=KRT74 PE=1 SV=2	2	4.16	1	1.32	1	1.32
P12277	KCRB_HUMAN Creatine kinase B-type OS=Homo sapiens GN=CKB PE=1 SV=1	6	24.67	8	26.51	5	16.8
P14618	KPYM_HUMAN Pyruvate kinase isozymes M1/M2 OS=Homo sapiens GN=PKM2 PE=1 SV=4	11	27.5	3	10.55	3	5.65
Q16363	LAMA4_HUMAN Laminin subunit alpha-4 OS=Homo sapiens GN=LAMA4 PE=1 SV=4	9	6.09	6	3.95	5	3.89
O15230	LAMA5_HUMAN Laminin subunit alpha-5 OS=Homo sapiens GN=LAMA5 PE=1 SV=8	9	3.06	5	1.6	10	3.46
P55268	LAMB2_HUMAN Laminin subunit beta-2 OS=Homo sapiens GN=LAMB2 PE=1 SV=2	28	17.85	18	13.57	21	13.79
P11047	LAMC1_HUMAN Laminin subunit gamma-1 OS=Homo sapiens GN=LAMC1 PE=1 SV=3	17	12.49	12	9.2	15	11.31
P00338	LDHA_HUMAN L-lactate dehydrogenase A chain OS=Homo sapiens GN=LDHA PE=1 SV=2	9	24.7	1	2.71	2	4.52
P07195	LDHB_HUMAN L-lactate dehydrogenase B chain OS=Homo sapiens GN=LDHB PE=1 SV=2	5	14.67	3	10.18	4	11.68
P09382	LEG1_HUMAN Galectin-1 OS=Homo sapiens GN=LGALS1 PE=1 SV=2	4	26.67	3	19.26	3	19.26
P17931	LEG3_HUMAN Galectin-3 OS=Homo sapiens GN=LGALS3 PE=1 SV=5	6	25.2	6	25.2	4	14.4
P02545	LMNA_HUMAN Prelamin-A/C OS=Homo sapiens GN=LMNA PE=1 SV=1	21	30.12	23	38.25	23	30.27
Q03252	LMNB2_HUMAN Lamin-B2 OS=Homo sapiens GN=LMNB2 PE=1 SV=3	3	4.67	3	3.83	8	14.5
Q93052	LPP_HUMAN Lipoma-preferred partner OS=Homo sapiens GN=LPP PE=1 SV=1	1	0.98	2	4.74	1	1.96
P51884	LUM_HUMAN Lumican OS=Homo sapiens GN=LUM PE=1 SV=2	3	12.43	10	35.5	8	23.96
P55145	MANF_HUMAN Mesencephalic astrocyte-derived neurotrophic factor OS=Homo sapiens GN=MANF PE=1 SV=3	1	8.24	1	8.24	1	8.24
P29966	MARCS_HUMAN Myristoylated alanine-rich C-kinase substrate OS=Homo sapiens GN=MARCKS PE=1 SV=4	6	30.72	5	21.08	5	21.08
P40926	MDHM_HUMAN Malate dehydrogenase, mitochondrial OS=Homo sapiens GN=MDH2 PE=1 SV=3	8	31.07	5	21.01	5	18.93
P20774	MIME_HUMAN Mimecan OS=Homo sapiens GN=OGN PE=1 SV=1	8	24.16	9	30.54	6	16.44
O14950	ML12B_HUMAN Myosin regulatory light chain 12B OS=Homo sapiens GN=MYL12B PE=1	3	18.6	9	50.58	7	48.84

	SV=2						
P26038	MOES_HUMAN Moesin OS=Homo sapiens GN=MSN PE=1 SV=3	7	11.27	5	8.32	2	2.77
P43121	MUC18_HUMAN Cell surface glycoprotein MUC18 OS=Homo sapiens GN=MCAM PE=1 SV=2	1	1.39	3	5.42	3	5.42
P35749	MYH11_HUMAN Myosin-11 OS=Homo sapiens GN=MYH11 PE=1 SV=3	76	33.67	108	46.3	87	36.87
P35579	MYH9_HUMAN Myosin-9 OS=Homo sapiens GN=MYH9 PE=1 SV=4	37	21.63	26	16.84	20	11.73
P24844	MYL9_HUMAN Myosin regulatory light polypeptide 9 OS=Homo sapiens GN=MYL9 PE=1 SV=4	2	12.21	10	50.58	8	49.42
P14543	NID1_HUMAN Nidogen-1 OS=Homo sapiens GN=NID1 PE=1 SV=3	13	11.87	9	9.46	12	10.91
Q02818	NUCB1_HUMAN Nucleobindin-1 OS=Homo sapiens GN=NUCB1 PE=1 SV=4	3	5.64	3	6.51	8	19.09
P36957	ODO2_HUMAN Dihydrolipoyllysine-residue succinyltransferase component of 2-oxoglutarate dehydrogenase complex, mitochondrial OS=Homo sapiens GN=DLST PE=1 SV=4	3	10.6	3	7.51	4	9.93
P68402	PA1B2_HUMAN Platelet-activating factor acetylhydrolase IB subunit beta OS=Homo sapiens GN=PAFAH1B2 PE=1 SV=1	1	3.93	1	3.93	1	3.93
Q99497	PARK7_HUMAN Protein DJ-1 OS=Homo sapiens GN=PARK7 PE=1 SV=2	1	13.76	5	30.16	4	16.4
Q15365	PCBP1_HUMAN Poly(rC)-binding protein 1 OS=Homo sapiens GN=PCBP1 PE=1 SV=2	2	6.74	1	3.09	1	3.09
P07237	PDIA1_HUMAN Protein disulfide-isomerase OS=Homo sapiens GN=P4HB PE=1 SV=3	11	22.64	8	13.58	16	32.48
Q9NR12-2	PDLI7_HUMAN Isoform 2 of PDZ and LIM domain protein 7 OS=Homo sapiens GN=PDLIM7	1	2.6	6	16.78	4	9.46
Q9NR12	PDLI7_HUMAN PDZ and LIM domain protein 7 OS=Homo sapiens GN=PDLIM7 PE=1 SV=1	1	2.41	6	13.79	4	8.75
P30086	PEBP1_HUMAN Phosphatidylethanolamine-binding protein 1 OS=Homo sapiens GN=PEBP1 PE=1 SV=3	4	26.74	6	41.71	5	37.43
P98160	PGBM_HUMAN Basement membrane-specific heparan sulfate proteoglycan core protein OS=Homo sapiens GN=HSPG2 PE=1 SV=4	18	4.94	25	8.24	17	4.76
O00264	PGRC1_HUMAN Membrane-associated progesterone receptor component 1 OS=Homo sapiens GN=PGRMC1 PE=1 SV=3	4	31.79	3	20.51	5	35.9
P35232	PHB_HUMAN Prohibitin OS=Homo sapiens	6	28.68	2	6.25	2	7.35

	GN=PHB PE=1 SV=1						
P15309	PPAP_HUMAN Prostatic acid phosphatase OS=Homo sapiens GN=ACPP PE=1 SV=3	3	11.14	7	18.91	8	20.98
P62937	PPIA_HUMAN Peptidyl-prolyl cis-trans isomerase A OS=Homo sapiens GN=PPIA PE=1 SV=2	4	18.79	5	29.7	4	18.79
P23284	PPIB_HUMAN Peptidyl-prolyl cis-trans isomerase B OS=Homo sapiens GN=PPIB PE=1 SV=2	5	24.07	6	26.85	4	20.83
Q06830	PRDX1_HUMAN Peroxiredoxin-1 OS=Homo sapiens GN=PRDX1 PE=1 SV=1	7	32.16	8	35.68	7	32.16
P32119	PRDX2_HUMAN Peroxiredoxin-2 OS=Homo sapiens GN=PRDX2 PE=1 SV=5	5	27.27	5	23.74	6	32.83
P30041	PRDX6_HUMAN Peroxiredoxin-6 OS=Homo sapiens GN=PRDX6 PE=1 SV=3	3	12.05	2	8.93	4	15.62
P51888	PRELP_HUMAN Prolargin OS=Homo sapiens GN=PRELP PE=1 SV=1	7	20.68	13	34.03	7	19.11
P07737	PROF1_HUMAN Profilin-1 OS=Homo sapiens GN=PFN1 PE=1 SV=2	4	40	6	45.71	4	40
Q6NZI2	PTRF_HUMAN Polymerase I and transcript release factor OS=Homo sapiens GN=PTRF PE=1 SV=1	3	8.97	3	11.28	2	8.46
Q3MIV8	Q3MIV8_HUMAN Myosin, heavy chain 11, smooth muscle OS=Homo sapiens GN=MYH11 PE=2 SV=1	75	34.26	106	46.34	85	36.74
Q3MNF0	Q3MNF0_HUMAN Myosin, heavy chain 11, smooth muscle OS=Homo sapiens GN=MYH11 PE=2 SV=1	75	34.5	106	46.53	85	36.97
Q3MNF1	Q3MNF1_HUMAN Myosin, heavy chain 11, smooth muscle OS=Homo sapiens GN=MYH11 PE=2 SV=1	76	33.91	108	46.49	87	37.09
Q6ZN40	Q6ZN40_HUMAN Tropomyosin 1 (Alpha), isoform CRA_f OS=Homo sapiens GN=TPM1 PE=2 SV=1	9	20.55	34	64.72	29	56.13
Q9Y427	Q9Y427_HUMAN Putative uncharacterized protein DKFZp586K2222 OS=Homo sapiens GN=DKFZp586K2222 PE=2 SV=2	8	22.18	31	66.2	26	56.34
P14927	QCR7_HUMAN Cytochrome b-c1 complex subunit 7 OS=Homo sapiens GN=UQCRB PE=1 SV=2	6	45.95	4	30.63	2	25.23
Q15293	RCN1_HUMAN Reticulocalbin-1 OS=Homo sapiens GN=RCN1 PE=1 SV=1	5	18.73	6	22.36	7	21.75
P26373	RL13_HUMAN 60S ribosomal protein L13 OS=Homo sapiens GN=RPL13 PE=1 SV=4	2	9	3	13.27	2	9
P05387	RLA2_HUMAN 60S acidic ribosomal protein	3	55.65	3	55.65	3	40.87

	P2 OS=Homo sapiens GN=RPLP2 PE=1 SV=1						
Q13151	ROA0_HUMAN Heterogeneous nuclear ribonucleoprotein A0 OS=Homo sapiens GN=HNRNPA0 PE=1 SV=1	1	5.25	1	10.49	1	5.25
P22626	ROA2_HUMAN Heterogeneous nuclear ribonucleoproteins A2/B1 OS=Homo sapiens GN=HNRNPA2B1 PE=1 SV=2	7	23.23	9	28.61	4	13.6
P04843	RPN1_HUMAN Dolichyl-diphosphooligosaccharide--protein glycosyltransferase subunit 1 OS=Homo sapiens GN=RPN1 PE=1 SV=1	6	13.18	1	2.31	1	1.81
P10301	RRAS_HUMAN Ras-related protein R-Ras OS=Homo sapiens GN=RRAS PE=1 SV=1	3	17.89	3	17.89	1	7.34
P62249	RS16_HUMAN 40S ribosomal protein S16 OS=Homo sapiens GN=RPS16 PE=1 SV=2	3	19.86	1	6.85	1	6.85
P62857	RS28_HUMAN 40S ribosomal protein S28 OS=Homo sapiens GN=RPS28 PE=1 SV=1	1	17.39	1	17.39	1	17.39
P62701	RS4X_HUMAN 40S ribosomal protein S4, X isoform OS=Homo sapiens GN=RPS4X PE=1 SV=2	1	3.42	1	3.42	1	3.42
P06703	S10A6_HUMAN Protein S100-A6 OS=Homo sapiens GN=S100A6 PE=1 SV=1	1	8.89	1	8.89	1	8.89
P31949	S10AB_HUMAN Protein S100-A11 OS=Homo sapiens GN=S100A11 PE=1 SV=2	3	23.81	3	23.81	2	17.14
P02743	SAMP_HUMAN Serum amyloid P-component OS=Homo sapiens GN=APCS PE=1 SV=2	7	31.39	6	27.8	6	27.8
O75368	SH3L1_HUMAN SH3 domain-binding glutamic acid-rich-like protein OS=Homo sapiens GN=SH3BGRL PE=1 SV=1	1	10.53	4	42.98	3	37.72
P54920	SNAA_HUMAN Alpha-soluble NSF attachment protein OS=Homo sapiens GN=NAPA PE=1 SV=3	1	5.08	1	5.08	1	5.08
P00441	SODC_HUMAN Superoxide dismutase [Cu-Zn] OS=Homo sapiens GN=SOD1 PE=1 SV=2	1	9.09	2	16.88	1	9.09
Q04837	SSBP_HUMAN Single-stranded DNA-binding protein, mitochondrial OS=Homo sapiens GN=SSBP1 PE=1 SV=1	4	33.11	1	12.16	1	10.14
O15061	SYNEM_HUMAN Synemin OS=Homo sapiens GN=SYNM PE=1 SV=2	6	4.47	32	24.22	26	19.42
O76070	SYUG_HUMAN Gamma-synuclein OS=Homo sapiens GN=SNCG PE=1 SV=2	1	11.02	2	24.41	1	11.02
Q01995	TAGL_HUMAN Transgelin OS=Homo sapiens GN=TAGLN PE=1 SV=4	6	25.37	17	55.22	8	37.81
Q9H853	TBA4B_HUMAN Putative tubulin-like protein alpha-4B OS=Homo sapiens GN=TUBA4B	1	5.81	2	10.79	2	10.79

	PE=5 SV=2						
P68371	TBB4B_HUMAN Tubulin beta-4B chain OS=Homo sapiens GN=TUBB4B PE=1 SV=1	5	13.71	6	15.96	4	12.13
P55072	TERA_HUMAN Transitional endoplasmic reticulum ATPase OS=Homo sapiens GN=VCP PE=1 SV=4	4	6.45	1	2.11	4	5.33
P49221	TGM4_HUMAN Protein-glutamine gamma-glutamyltransferase 4 OS=Homo sapiens GN=TGM4 PE=1 SV=2	7	12.43	18	37.43	4	7.46
P24752	THIL_HUMAN Acetyl-CoA acetyltransferase, mitochondrial OS=Homo sapiens GN=ACAT1 PE=1 SV=1	2	7.03	2	7.03	2	6.79
Q9Y490	TLN1_HUMAN Talin-1 OS=Homo sapiens GN=TLN1 PE=1 SV=3	8	4.68	34	19.99	23	12.67
P60174	TPIS_HUMAN Triosephosphate isomerase OS=Homo sapiens GN=TPI1 PE=1 SV=3	6	26.22	7	28.32	6	26.22
P07951-2	TPM2_HUMAN Isoform 2 of Tropomyosin beta chain OS=Homo sapiens GN=TPM2	13	33.8	32	72.89	26	62.32
P07951-3	TPM2_HUMAN Isoform 3 of Tropomyosin beta chain OS=Homo sapiens GN=TPM2	9	27.82	22	63.31	17	54.84
P67936-2	TPM4_HUMAN Isoform 2 of Tropomyosin alpha-4 chain OS=Homo sapiens GN=TPM4	10	25.7	23	51.06	14	35.56
P67936	TPM4_HUMAN Tropomyosin alpha-4 chain OS=Homo sapiens GN=TPM4 PE=1 SV=3	12	31.45	22	52.02	15	42.74
P02787	TRFE_HUMAN Serotransferrin OS=Homo sapiens GN=TF PE=1 SV=3	2	2.58	10	13.47	4	6.59
P07996	TSP1_HUMAN Thrombospondin-1 OS=Homo sapiens GN=THBS1 PE=1 SV=2	6	6.58	1	1.03	1	0.85
P21796	VDAC1_HUMAN Voltage-dependent anion-selective channel protein 1 OS=Homo sapiens GN=VDAC1 PE=1 SV=2	7	36.75	1	7.07	3	15.55
P08670	VIME_HUMAN Vimentin OS=Homo sapiens GN=VIM PE=1 SV=4	12	26.18	19	39.06	16	31.97
P18206	VINC_HUMAN Vinculin OS=Homo sapiens GN=VCL PE=1 SV=4	13	14.29	22	24.07	18	16.58
P04004	VTNC_HUMAN Vitronectin OS=Homo sapiens GN=VTN PE=1 SV=1	3	7.95	1	3.14	1	2.51
Q6PCB0	VWA1_HUMAN von Willebrand factor A domain-containing protein 1 OS=Homo sapiens GN=VWA1 PE=2 SV=1	3	9.44	5	17.08	3	9.44
Q96MR6	WDR65_HUMAN WD repeat-containing protein 65 OS=Homo sapiens GN=WDR65 PE=1 SV=3	1	0.88	1	0.88	1	0.88
P13010	XRCC5_HUMAN X-ray repair cross-complementing protein 5 OS=Homo sapiens	3	4.78	1	1.64	2	3.28

	GN=XRCC5 PE=1 SV=3						
P25311	ZA2G_HUMAN Zinc-alpha-2-glycoprotein OS=Homo sapiens GN=AZGP1 PE=1 SV=2	3	15.1	6	24.16	8	29.19

Supplemental Table 2. List of proteins found in PrEC only, PrEC with Patient 18 EVs, and PrEC with Patient 19 EVs.

Accession #	Protein Name	PrEC only		PrEC + P18 EVs		PrEC +19 EVs	
		# Unique Peptides	% Sequence Coverage	# Unique Peptides	% Sequence Coverage	# Unique Peptides	% Sequence Coverage
P62258	1433E_HUMAN 14-3-3 protein epsilon OS=Homo sapiens GN=YWHAE PE=1 SV=1	2	7.31	2	7.82	2	3.82
A8K092	A8K092_HUMAN ATP synthase subunit alpha OS=Homo sapiens GN=ATP5A1 PE=2 SV=1	0	0	8	15.11	4	8.55
O43707	ACTN4_HUMAN Alpha-actinin-4 OS=Homo sapiens GN=ACTN4 PE=1 SV=2	0	0	5	5.82	4	5.27
P04075	ALDOA_HUMAN Fructose-bisphosphate aldolase A OS=Homo sapiens GN=ALDOA PE=1 SV=2	2	5.22	1	3.3	2	7.14
P04083	ANXA1_HUMAN Annexin A1 OS=Homo sapiens GN=ANXA1 PE=1 SV=2	5	13.58	9	28.9	10	28.9
P06576	ATPB_HUMAN ATP synthase subunit beta, mitochondrial OS=Homo sapiens GN=ATP5B PE=1 SV=3	2	3.97	10	28.36	11	30.43
P48047	ATPO_HUMAN ATP synthase subunit O, mitochondrial OS=Homo sapiens GN=ATP5O PE=1 SV=1	0	0	1	5.16	0	0
B4E022	B4E022_HUMAN Uncharacterized protein OS=Homo sapiens GN=TKT PE=2 SV=1	0	0	5	10.94	4	6.25
P00918	CAH2_HUMAN Carbonic anhydrase 2 OS=Homo sapiens GN=CA2 PE=1 SV=2	0	0	0	0	1	3.46
P27797	CALR_HUMAN Calreticulin OS=Homo sapiens GN=CALR PE=1 SV=1	0	0	1	1.68	2	4.56
P27824	CALX_HUMAN Calnexin OS=Homo sapiens GN=CANX PE=1 SV=2	0	0	5	7.43	1	1.69
P10809	CH60_HUMAN 60 kDa heat shock protein, mitochondrial OS=Homo sapiens GN=HSPD1 PE=1 SV=2	0	0	6	12.39	5	10.65
P00403	COX2_HUMAN Cytochrome c oxidase subunit 2 OS=Homo sapiens GN=MT-CO2 PE=1 SV=1	3	4.11	4	5.02	4	4.97
Q08211	DHX9_HUMAN ATP-dependent RNA helicase A OS=Homo sapiens GN=DHX9 PE=1 SV=4	0	0	0	0	0	0

E7EX29	E7EX29_HUMAN Tyrosine 3-monooxygenase/tryptophan 5-monooxygenase activation protein, zeta polypeptide (Fragment) OS=Homo sapiens GN=YWHAZ PE=3 SV=1	3	11.38	1	3.25	1	3.25
P68104	EF1A1_HUMAN Elongation factor 1-alpha 1 OS=Homo sapiens GN=EEF1A1 PE=1 SV=1	8	17.53	8	16.45	8	16.45
P13639	EF2_HUMAN Elongation factor 2 OS=Homo sapiens GN=EEF2 PE=1 SV=4	0	0	7	7.58	5	4.78
P06733	ENOA_HUMAN Alpha-enolase OS=Homo sapiens GN=ENO1 PE=1 SV=2	4	8.06	5	11.06	2	3.92
P14625	ENPL_HUMAN Endoplasmin OS=Homo sapiens GN=HSP90B1 PE=1 SV=1	0	0	11	12.95	8	9.84
F8VZY9	F8VZY9_HUMAN Keratin 18 OS=Homo sapiens GN=KRT18 PE=3 SV=1	3	6.14	2	4.09	6	12.79
P04406	G3P_HUMAN Glyceraldehyde-3-phosphate dehydrogenase OS=Homo sapiens GN=GAPDH PE=1 SV=3	5	16.72	5	14.93	3	10.15
P11021	GRP78_HUMAN 78 kDa glucose-regulated protein OS=Homo sapiens GN=HSPA5 PE=1 SV=2	1	2.14	25	41.59	15	27.06
P09211	GSTP1_HUMAN Glutathione S-transferase P OS=Homo sapiens GN=GSTP1 PE=1 SV=2	0	0	0	0	0	0
H0YG33	H0YG33_HUMAN Heat shock 70kDa protein 1B OS=Homo sapiens GN=HSPA1B PE=3 SV=1	0	0	3	4.8	2	3.53
P62805	H4_HUMAN Histone H4 OS=Homo sapiens GN=HIST1H4A PE=1 SV=2	7	51.46	4	38.83	0	0
P07900	HS90A_HUMAN Heat shock protein HSP 90-alpha OS=Homo sapiens GN=HSP90AA1 PE=1 SV=5	0	0	9	11.61	5	7.79
P08238	HS90B_HUMAN Heat shock protein HSP 90-beta OS=Homo sapiens GN=HSP90AB1 PE=1 SV=4	1	0.97	16	22.38	16	19.06
P11142	HSP7C_HUMAN Heat shock cognate 71 kDa protein OS=Homo sapiens GN=HSPA8 PE=1 SV=1	0	0	13	22.6	6	12.69
P13645	K1C10_HUMAN Keratin, type I cytoskeletal 10 OS=Homo sapiens GN=KRT10 PE=1 SV=6	5	7.02	12	19.52	14	26.71
P02533	K1C14_HUMAN Keratin, type I cytoskeletal 14 OS=Homo sapiens GN=KRT14 PE=1 SV=4	4	6.78	5	7.2	6	7.84
Q04695	K1C17_HUMAN Keratin, type I cytoskeletal 17 OS=Homo sapiens GN=KRT17 PE=1 SV=2	3	5.32	4	5.79	5	7.18
P08727	K1C19_HUMAN Keratin, type I cytoskeletal 19 OS=Homo sapiens GN=KRT19 PE=1 SV=4	4	8	5	8.5	6	10.5
P35527	K1C9_HUMAN Keratin, type I cytoskeletal 9 OS=Homo sapiens GN=KRT9 PE=1 SV=3	6	8.67	4	6.26	10	13.64

P35908	K22E_HUMAN Keratin, type II cytoskeletal 2 epidermal OS=Homo sapiens GN=KRT2 PE=1 SV=2	4	4.54	11	15.34	15	25.98
P04264	K2C1_HUMAN Keratin, type II cytoskeletal 1 OS=Homo sapiens GN=KRT1 PE=1 SV=6	9	13.98	18	26.4	20	29.04
P13647	K2C5_HUMAN Keratin, type II cytoskeletal 5 OS=Homo sapiens GN=KRT5 PE=1 SV=3	2	1.53	4	5.25	4	5.25
P04259	K2C6B_HUMAN Keratin, type II cytoskeletal 6B OS=Homo sapiens GN=KRT6B PE=1 SV=5	3	3.37	6	8.87	5	7.09
P08729	K2C7_HUMAN Keratin, type II cytoskeletal 7 OS=Homo sapiens GN=KRT7 PE=1 SV=5	2	1.92	3	3.62	2	1.92
P78386	KRT85_HUMAN Keratin, type II cuticular Hb5 OS=Homo sapiens GN=KRT85 PE=1 SV=1	0	0	0	0	0	0
P07195	LDHB_HUMAN L-lactate dehydrogenase B chain OS=Homo sapiens GN=LDHB PE=1 SV=2	4	11.38	2	4.19	2	5.39
P14174	MIF_HUMAN Macrophage migration inhibitory factor OS=Homo sapiens GN=MIF PE=1 SV=4	1	9.57	0	0	0	0
P26038	MOES_HUMAN Moesin OS=Homo sapiens GN=MSN PE=1 SV=3	0	0	3	4.51	3	3.99
P35579	MYH9_HUMAN Myosin-9 OS=Homo sapiens GN=MYH9 PE=1 SV=4	0	0	2	1.12	0	0
P13667	PDIA4_HUMAN Protein disulfide-isomerase A4 OS=Homo sapiens GN=PDIA4 PE=1 SV=2	0	0	5	8.53	1	1.55
P30086	PEBP1_HUMAN Phosphatidylethanolamine-binding protein 1 OS=Homo sapiens GN=PEBP1 PE=1 SV=3	4	15.21	5	25.76	4	15.11
P35232	PHB_HUMAN Prohibitin OS=Homo sapiens GN=PHB PE=1 SV=1	3	11.76	2	8.46	2	8.09
P62937	PPIA_HUMAN Peptidyl-prolyl cis-trans isomerase A OS=Homo sapiens GN=PPIA PE=1 SV=2	4	29.09	0	0	0	0
P23284	PPIB_HUMAN Peptidyl-prolyl cis-trans isomerase B OS=Homo sapiens GN=PPIB PE=1 SV=2	1	3.7	0	0	1	3.7
Q06830	PRDX1_HUMAN Peroxiredoxin-1 OS=Homo sapiens GN=PRDX1 PE=1 SV=1	6	29.15	6	27.64	9	45.73
P30041	PRDX6_HUMAN Peroxiredoxin-6 OS=Homo sapiens GN=PRDX6 PE=1 SV=3	0	0	0	0	0	0
P07737	PROF1_HUMAN Profilin-1 OS=Homo sapiens GN=PFN1 PE=1 SV=2	2	14.29	0	0	0	0
P22695	QCR2_HUMAN Cytochrome b-c1 complex subunit 2, mitochondrial OS=Homo sapiens GN=UQCRC2 PE=1 SV=3	0	0	1	3.09	0	0

P30050	RL12_HUMAN 60S ribosomal protein L12 OS=Homo sapiens GN=RPL12 PE=1 SV=1	1	5.45	1	5.45	0	0
P35268	RL22_HUMAN 60S ribosomal protein L22 OS=Homo sapiens GN=RPL22 PE=1 SV=2	0	0	0	0	1	4.69
P61353	RL27_HUMAN 60S ribosomal protein L27 OS=Homo sapiens GN=RPL27 PE=1 SV=2	1	6.62	0	0	0	0
P62269	RS18_HUMAN 40S ribosomal protein S18 OS=Homo sapiens GN=RPS18 PE=1 SV=3	3	17.11	0	0	0	0
P39019	RS19_HUMAN 40S ribosomal protein S19 OS=Homo sapiens GN=RPS19 PE=1 SV=2	2	11.72	0	0	1	4.83
P62701	RS4X_HUMAN 40S ribosomal protein S4, X isoform OS=Homo sapiens GN=RPS4X PE=1 SV=2	0	0	0	0	0	0
P37802	TAGL2_HUMAN Transgelin-2 OS=Homo sapiens GN=TAGLN2 PE=1 SV=3	1	6.03	1	4.02	0	0
P68363	TBA1B_HUMAN Tubulin alpha-1B chain OS=Homo sapiens GN=TUBA1B PE=1 SV=1	2	5.54	9	25.28	5	13.53
P60174	TPIS_HUMAN Triosephosphate isomerase OS=Homo sapiens GN=TPI1 PE=1 SV=3	1	4.55	0	0	1	4.55
P22314	UBA1_HUMAN Ubiquitin-like modifier-activating enzyme 1 OS=Homo sapiens GN=UBA1 PE=1 SV=3	0	0	0	0	0	0
P21796	VDAC1_HUMAN Voltage-dependent anion-selective channel protein 1 OS=Homo sapiens GN=VDAC1 PE=1 SV=2	1	4.95	2	7.07	1	4.95
